# Mapping groundwater potential zone in the subarnarekha basin, India, using a novel hybrid multi-criteria approach in Google earth Engine

**DOI:** 10.1016/j.heliyon.2024.e24308

**Published:** 2024-01-07

**Authors:** Chiranjit Singha, Kishore Chandra Swain, Biswajeet Pradhan, Dinesh Kumar Rusia, Armin Moghimi, Babak Ranjgar

**Affiliations:** a Department of Agricultural Engineering, Institute of Agriculture, Visva-Bharati (A Central University), Sriniketan, 731236, West Bengal, India; b Centre for Advanced Modelling and Geospatial Information Systems (CAMGIS), School of Civil and Environmental Engineering, Faculty of Engineering and IT, University of Technology Sydney, Sydney, NSW 2007, Australia; c Earth Observation Centre, Institute of Climate Change, Universiti Kebangsaan Malaysia, Bangi 43600 UKM, Selangor, Malaysia; d Ludwig-Franzius-Institute for Hydraulic, Estuarine and Coastal Engineering, Leibniz University Hannover, Nienburger Str. 4, 30167 Hanover, Germany; e Department of Energy, Politecnico di Milano, Via Privata Giuseppe La Masa, 34, 20156, Milan, Italy

**Keywords:** GWPZ, Hydrologic, Normalized difference vegetation index, FuzzyDEMATEL, Random forest, Multi-collinearity

## Abstract

Assessing groundwater potential for sustainable resource management is critically important. In addressing this concern, this study aims to advance the field by developing an innovative approach for Groundwater potential zone (GWPZ) mapping using advanced techniques, such as FuzzyAHP, FuzzyDEMATEL, and Logistic regression (LR) models. GWPZ was carried out by integrating various primary factors, such as hydrologic, soil permeability, morphometric, terrain distribution, and anthropogenic influences, incorporating twenty-seven individual criteria using multi-criteria decision models along with a hybrid approach for the Subarnarekha River basin, India, in Google earth engine (GEE). The predictive capability of the model was evaluated using a Multi-Collinearity test (VIF <10.0), followed by applying a random forest model, considering the weighted impact of the five primary factors. The hybrid model for GWPZ classification showed that 21.97 % (4256.3 km^2^) of the area exhibited very high potential, while 11.37 % (2202.1 km^2^) indicated very low potential for GW in this area. Validation of the groundwater level data from 72 observation wells, performed by the Area under receiver operating characteristic (AUROC) curve technique, yielded values ranging between 75 % and 78 % for different models, underscoring the robust predictability of GWPZ. The hybrid and LR-FuzzyAHP models demonstrated remarkable effectiveness in GWPZ mapping, indicating that the downstream and southern regions boast substantial groundwater potential attributed to alluvial soil and favorable recharge conditions. Conversely, the central part grapples with a scarcity of groundwater. It holds the potential to assist planners and managers in formulating strategies for managing groundwater levels and alleviating the impacts of future droughts.

## 1 Introduction

As the most dynamic and sustainable renewable natural resource, groundwater both maintains the Earth's biogeochemical cycle equilibrium [1] and serves as a viable alternative water source in dry and semi-dry areas globally [2]. It is a prominent source of fresh drinking water for human consumption, with many developing countries depending heavily on it for various purposes, including agriculture, domestic usage, and urban and industrial growing [3]. The water demand is steadily rising as a consequence of population expansion, urban expansion, industrialization, and agricultural irrigation, potentially hurting groundwater storage and quality [4]. Unregulated groundwater development can lead to water scarcity issues, posing challenges in addressing environmental degradation and climate change patterns [5,6]. Therefore, groundwater management becomes vital for soil conservation efforts to ensure future food security.

In India, groundwater is pivotal in Gross domestic product (GDP) growth. It accounts for 50 % of water consumption in urban areas and more than 80 % in rural areas [7]. The Central Groundwater Board [8] reported that India's annual renewable groundwater resource is ∼433 Billion cubic meters (BCM). The primary groundwater consumption is in the irrigation sector, accounting for 92 % (213 BCM), followed by industrial and domestic use, which accounts for 18 BCM. Groundwater monitoring is essential for estimating river basins' water budgets, enabling sustainable hydrologic decision support systems. It also facilitates water quality monitoring for local livelihoods [9]. India has several river basins, including the Ganga, Brahmaputra, Godabari, Narmada, Mahanadi, and Suvarnarekha, among others.

The Suvarnarekha River basin is of significant importance in meeting the urban water demands, agriculture, irrigation needs, hydroelectric power generation, domestic use, and industrial requirements in the states of Jharkhand, Odisha, and even West Bengal. During the 2000–2010 period, the annual average groundwater recharge rate in the river basin varied from 519 mm to 858 mm [10]. However, in recent decades, the ecological environment of the Subarnarekha catchment area has deteriorated due to various factors, including low-intensity rainfall, reduced water holding capacity, increased mining activity, industrial growth, soil erosion, deforestation, and water pollution [[11], [12], [13]]. According to research conducted by Gautam et al. [14], heavy metals and nitrate pollution have been the main drivers behind the reduction in groundwater quality across the entire catchment area. Monitoring groundwater quantity and quality is urgently needed for sustainable development in the whole basin, as groundwater is less susceptible to contamination compared to surface water [15]. Mandal et al. [10] also experienced a significant decrease in the recharge rate, particularly for the years 2000, 2002, and 2009, which calls for special attention.

The quantification and delineation of groundwater resources employing conventional techniques such as geological, geophysical, or hydrogeological methods are often labor-intensive, cost-ineffective, and time-consuming [16]. Therefore, recording and evaluating the outcomes of subsurface hydrological inquiries can provide a better alternative approach to traditional groundwater potential mapping. A cohesive method combining Remote sensing (RS) and Geographic information system (GIS) approach can serve as a superior decision support system for intelligently assessing Groundwater Potential (GWP), groundwater quality suitability, discharge, recharge, and storage mapping [[17], [18], [19], [20], [21]]. Mohammed et al. [[22], [23]] integrated RS and GIS techniques with the Analytic hierarchy process (AHP) method for effectively evaluating potential groundwater recharge zones in the Iraqi Western desert region. Tamesgen et al. [24] presented the GWP analysis with nine geo-environmental parameters through Ethiopia's RS/GIS-based Multi-Criteria Decision Making (MCDM) approach. Kisiki et al. [25]used geospatial and RS data to define the groundwater recharge zones through the GWP evaluation. They then performed a sensitivity analysis to determine the impact of hydrologic and geological factors on their variations. The widely used multi-criteria-based decision support techniques for GWP mapping include AHP [22,[26], [27], [28]], Frequency ratio (FR) [29], Logistic regression (10.13039/501100009319LR) [30], Fuzzy set [31], Quick unbiased efficient statistical tree (QUEST) [32], Weighted linear combination (WLC) [33], Evidential belief function (EBF) [34], Multi-influencing factor (10.13039/501100001699MIF) [35], Shannon's entropy [36], TOPSIS [37], Dempster-Shafer model [38], Bayesian network model [39] etc. Causal relationships based on Fuzzy decision-making trial and evaluation laboratory (FDEMATEL) approaches have also been applied for soil erosion, flood, and landslide susceptibility mapping [40,41]. Such integrated methods have also been used for groundwater potential mapping, for instance the study by Echogdali [42] in the Akka Basin, Morocco.

Recent trends showed a paradigm shift towards developing big data geospatial hybrid MCDM-based cloud environments that enabled machine learning models for mapping groundwater productivity and availability. Emerging cloud computing platforms, including Google earth engine (GEE), Climate Engine (CE), SEPAL, IBM Cloud, OpenEO, Amazon web services (AWS), and Microsoft Azure cloud technology, have shown their capability in handling a vast amount of Analysis-ready product (ARP) conditioning parameters. These platforms effectively expedite decision-making, surpassing traditional data pre-processing methods [[43], [44], [45], [46], [47]]. Al-Ozeer et al. [48] used Azure Cloud with many parameters to quantify groundwater potential maps in Northern Iraq. The integration of RS, cloud computing, and MCDM with a AHP architecture has ushered in trends in achieving high efficiency in groundwater potential mapping [49].

GEE is an open-source, freely distributed cloud platform with petabyte-level storage ability, having earth observation data over the past four decades [[50], [51], [52]]. Magnoni et al. [53] incorporated GEE cloud computing with hydrological FLDAS models to identify groundwater dynamics in the suitability area for groundwater recharge zones during 2014–2017 in Brazil. Previous research had been limited to topography and a few conditioning parameters for quantifying GWP mapping. However, the new GEE cloud provides more parameters to aid groundwater assessment, evaluation, and conservation [54]. carried out GWP zoning using the GEE platform, integrating GIS and RS techniques by incorporating fifteen groundwater recharge monitoring parameters for Islamabad, Pakistan. They identified the 15 % area as suitable for extracting groundwater.

While conventional methods of mapping and delineating GWPZ often rely on ground field measures/surveys and costly hydrogeological and geophysical tools, the appearance of the GEE cloud presents a transformative opportunity. GEE provides extensive access to many conditioning parameters, including hydrological, permeability, morphometric, and anthropogenic criteria, enabling real-time GWPZ mapping with ample storage capacity. Nevertheless, in the context of the Subarnarekha River basin, limited studies have been conducted to ascertain the potential occurrence of subterranean water. Bridging this research gap, integrating cloud-based Geotools presents a affordable and expeditious means of generating and modeling geoscientific data. This current study used a synthesis of MCDM processes with GIS and RS models to outline groundwater potential areas.

The study's novelty includes incorporating a large number of influencing factors for GW potential zone mapping through various multi-criteria analysis approaches enabled by the GEE cloud platform. The study also indicated that the combination of MCDM variation and RS-GIS for groundwater prospecting could be a cost-effective technique that could overcome the limitations of traditional methods. The study also encouraged the utilization of secondary information for quick assessment of GWPZ. In addition, this study provides deep insight into the ground storage change analysis in specific periods. The database can be utilized by the officials and authorities to adequately plan and manage the artificial recharge projects in the study area, ensuring that the region's consumption stays sustainable. The scope of this study extends to near-real-time monitoring of underground water potential within any given watershed. The insights from this research will be instrumental in formulating a sustainable groundwater management strategy. Additionally, it will serve as a valuable resource for both private and public sectors in identifying optimal locations for borehole drilling operations.

The study was carried out to GWPZ by using three MCDM approaches, namely, Fuzzy Analytic Hierarchy Process (FAHP), Fuzzy Decision-Making Trial and Evaluation Laboratory (FDEMATEL), and Logistic Regression (LR) with the assistance of twenty-seven influential parameters in the GEE cloud platform. Additionally, data from 72 well locations were incorporated to detect appropriate zones for groundwater recharge.

## 2 Materials and methods

### 2.1 Study area

**Fig. 1:**
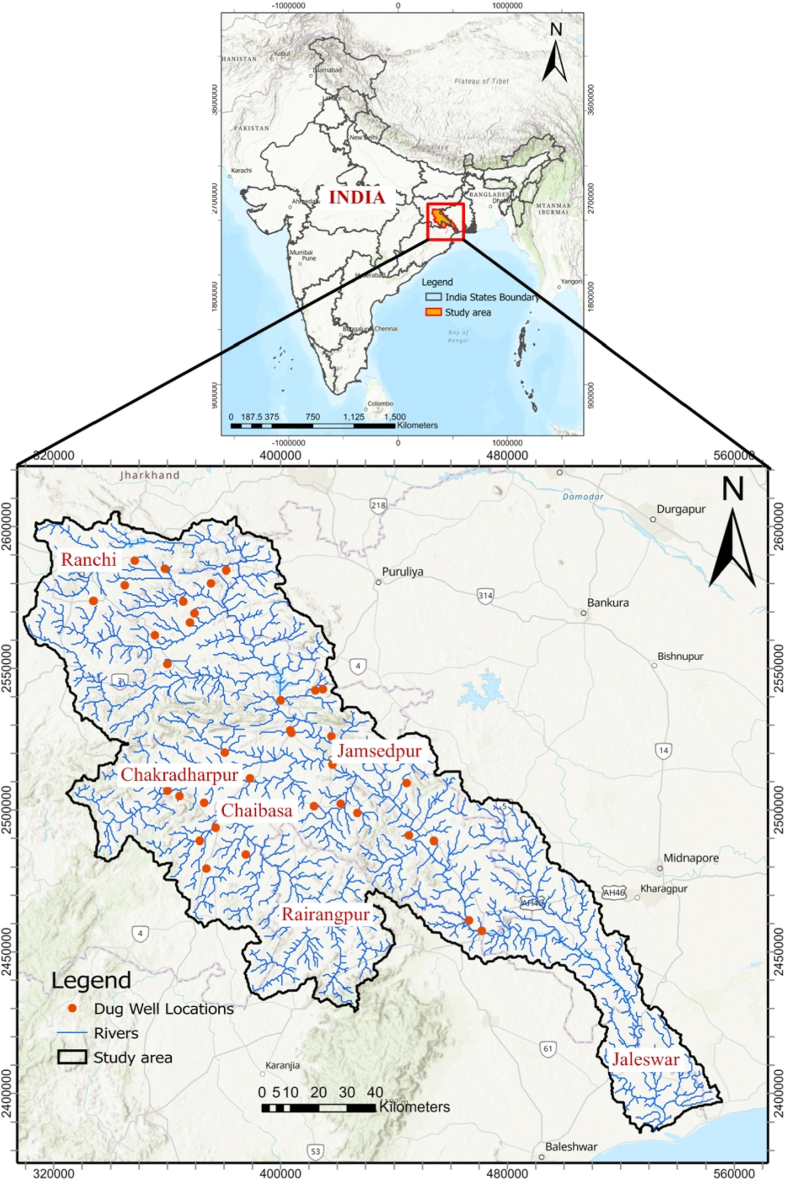
Study area map.

The Subarnarekha River basin, located in the Jharkhand region of India (Fig. 1), covers an area of 19,296 km2. The study area is positioned between the latitude of 21° 33′ to 23°32′ East and longitude of 85° 09′ to 87° 27’ North, with an elevation of 740 m in a heterogeneous landscape. The fundamental landscape portion of our study area in its eastern side is marked by river terraces with newer geological formations, such as tertiary gravels, recently deposited alluvium, and Pleistocene alluvium [12]. Diverse parent materials and various soil groups, such as alluvial, red, and latosol soils, underline the river basin area. The basin area experiences a humid tropical climate characterized by hot summers and mild winters with an average annual rainfall of 1400 mm. According to Jain et al. (2007), around 80–90 % of total annual precipitation occurs during June–October. The basin has 12 gauge discharge sites and two flood forecasting stations. Moreover, the Subarnarekha basin provides the three states (West Bengal, Odisha, Jharkhand) with a considerable water source for their industrial, irrigation, and municipal needs.

This basin include a variety of mineral materials (e.g. copper, iron, gold, and uranium), which can be exposed as a result of unplanned mining as well as untreated industrial domestic wastewater, thus polluting the river and threatening marine life [55]. Heavy rainfall in *the Chhotonagpur* plateau generally brings floods and heavy siltation in the lower Subarnarekha basin, causing loss of property, domestic animals, and sometimes people.

There are 38 dams (i.e., Hatia, Getasuld, Galudih, Chandil), 12 barrages, and four weirs in the river basin for providing irrigation in the surrounding region. The part of the Subarnarekha basin shows more than a 4 m rise in groundwater level fluctuation due to recharge and fall of less than 2 m in most of its parts. Higher levels of chlorine (up to 85.2 mg/L) and sodium (up to 39 mg/L) are present in groundwater [11].

### 2.2 Groundwater inventory mapping

The groundwater inventory data were acquired from the Central Ground Water Board, India [8]. Groundwater wells, with a high yield of ≥10 m^3^h^-1^ (mean of score) were considered as a threshold for potentiality GWPZ activity in our study area. For cross-validation, 72 well were used as samples to produce the GWPZ through a training (70 %) and testing (30 %) validation approach.

### 2.3 Methodology

**Fig. 2:**
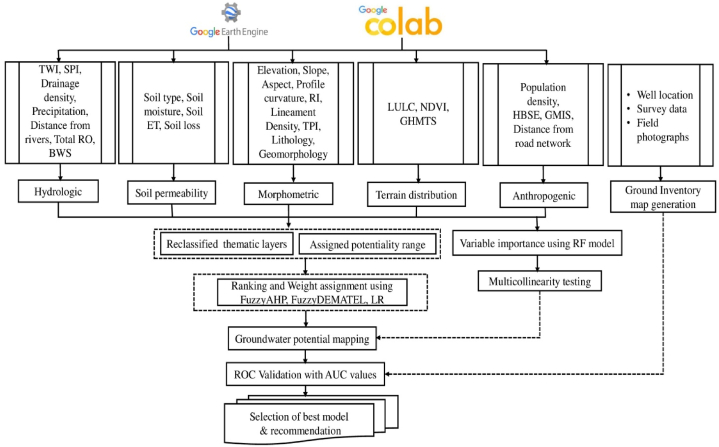
Details workflow of the steps for the GWPZs mapping.

The GWPZ mapping methodology was established before fieldwork, as illustrated in Fig. 2. This comprehensive framework covers five distinct stages. Firstly, it involves the preparation of the GWPZ conditioning database. Following this, sufficient data is employed to create Groundwater inventory mapping. The third stage incorporates applying the Random Forest (RF) model and Multi-collinearity analysis to determine variable importance and select GWPZ conditioning factors. Subsequently, spatial predictions for GWPZ maps are generated using a combination of FuzzyAHP, FuzzyDEMATEL, Logistic Regression (LR), and hybrid models. Finally, a feature selection analysis is executed employing the Boruta technique. The resulting maps are then rigorously validated using the Area Under the Curve (AUC) curve [39] to determine the most effective model for GWPZ mapping.

### 2.4 Source of data

The present research demonstrates using numerous factors for groundwater potential assessment in the Subarnarekha River basin. Five main factors were selected through an broad investigation of the literature and expert opinions on their relevance to groundwater assessment. These twenty-seven sub-factors were designated based on the relative influence of each data layer, determined through practical experience and knowledge of factors affecting GWPZ mapping. They were then adjusted based on their proximity to specific sub-categories [54]. The five major conditioning factors chosen are hydrological criteria, morphometric criteria, permeability, terrain distribution, and anthropogenic factors (see Table 1 and Supplementary Fig. 1).

**Table 1:** Data layers sources and description for groundwater potential mapping.

Sl no.	Data type	Description	factors	Source
1	DEM	SRTM (30 m)	Elevation, slope, aspect, Profile curvature, TWI, SPI, TPI, Ruggedness index	USGS/GEE
2	Geomorphology	Geological Survey of India (GSI 2019), 1:250,000	Geomorphology	http://bhukosh.gsi.gov.in/Bhukosh
3	Lithology	Geological Survey of India (GSI 2019), 1:250,000	Lithology	http://bhukosh.gsi.gov.in/Bhukosh
	Lineament	Geological Survey of India (GSI 2019), 1:250,000	Lineament density	http://bhukosh.gsi.gov.in/Bhukosh
4	Soil data	Harmonized World Soil Database v 1.2, 30 arc-second	Soil texture	http://www.fao.org/soils-portal/soil-survey/soil-maps-and-databases/harmonized-world-soil-database-v12/en/
5	Soil moisture	NASA_USDA/HSL/SMAP_soil_moisture, ssm band, 0.25°	Soil moisture	NASA GSFC/GEE
6	Soil loss (t ha-1 yr-1)	Global Soil Erosion Modelling platform (GloSEM)version 1.1, Resampled 250 m,	Soil loss	https://esdac.jrc.ec.europa.eu/content/global-soil-erosion
7	Precipitation (mm/yearly)	UCSB-CHG/CHIRPS/PENTAD (CHIRPS Pentad: Climate Hazards Group InfraRed Precipitation with Station Data (version 2.0 final), 0.05°	Precipitation	https://code.earthengine.google.com/
8	Baseline water stress	Aqueduct 3.0: Updated Decision-Relevant Global Water Risk Indicators (Aqueduct Water Risk Atlas), 5 × 5 arc minutes	Water stress	https://www.wri.org/publication/aqueduct-30
9	Evapotranspiration	Time Averaged Map of Evapotranspiration (daily 0.25 deg. [GLDAS Model GLDAS_CLSM025_DA1_D v2.2] kg m-2 s-1) 2010–2020,	Evapotranspiration	https://giovanni.gsfc.nasa.gov/giovanni
10	Total Runoff	Time Averaged Map of Strom surface & Baseflow-groundwater runoff daily 0.25 deg. [GLDAS Model GLDAS_CLSM025_DA1_D v2.2] kg m-2 s-1), 2003-02-01 - 2020-01-01	Runoff	https://giovanni.gsfc.nasa.gov/giovanni
11	Land use/land cover	ESA/WorldCover/v100, 2020),10 m	Land use/land cover	https://code.earthengine.google.com/
12	Sentinel 2 MSI Images	COPERNICUS/S2 level-1C, (2019-01-01 - 2019–12-31), 10 m	NDVI	https://code.earthengine.google.com/
13	Population density	CIESIN/GPWv411/GPW_Population_Density, 30 arc seconds	Population density	NASA Socioeconomic Data and Applications Center(SEDAC). https://code.earthengine.google.com
14	GHMTS	The Global Human Modification of Terrestrial Systemsv1 (2016), 1-km	GHMTS	https://sedac.ciesin.columbia.edu/data/set/lulc-human-modification-terrestrial-systems
15	GMIS	Global Man-made Impervious Surface (GMIS) Dataset From Landsat, v1, 30 m	GMIS	https://sedac.ciesin.columbia.edu/data/set/ulandsat-gmis-v1
16	HBSE	Global Human Built-up And Settlement Extent (HBASE) Dataset From Landsat, v1, 30 m	HBSE	https://sedac.ciesin.columbia.edu/data/set/ulandsat-hbase-v1
17	Road network	Road network in Subarnarekha basin region	Distance from roads	https://www.openstreetmap.org/export#map=6/22.431/83.035
18	River network	WWF HydroSHEDS Free-Flowing Rivers Network v1(15 arc-seconds)	Drainage density, Distance from rivers	USGS/GEE

### 2.5 Groundwater potential evaluation

Various geo-environmental factors play a significant role in determining the groundwater status of an area. In this study, conditioning factors were used for GWPZ mapping (Table 2). A total of twenty-seven primary Groundwater conditioning factors (GWCFs) were classified into five major groups based on their similar properties and consistency (Supplementary Fig. 1). Those conditioning factors are: I): Hydrologic factors: Topographic wetness index (TWI), Stream power index (SPI), Drainage density, Precipitation, Distance from rivers, Total Run-Off (RO), and Groundwater stress, II): Permeability factors: Soil type, Soil moisture, Soil evapotranspiration (ET) and Soil loss III): Morphometric factors: Elevation, Slope, Aspect, Profile curvature, Ruggedness index, Lineament density, Topographic position index, Lithology, Geomorphology IV) Terrain distribution factors: Land use/land cover, Normalized differential vegetation index (NDVI), The Global Human Modification of Terrestrial System (GHMTS) and; V) Anthropogenic factors: Population density, Global Human Built-up and Settlement Extent (HBASE), Global Man-made Impervious Surface (GMIS), and Distance from roads [[42], [45], [54]]

**Table 2:** Selected GWPZs criterion range with five potentiality classes.

Criterion	Sub-criterion	Level	Very Highly (5)	Highly (4)	Moderately (3)	Low (2)	Very Low (1)
(A) Morphometric							
1	Elevation	(m)	0–20	21–36	37–53	54–74	>75
2	Slope	0	0–2.4	2.4–5.5	5.5–10.7	10.7–19.7	19.7–54.27
3	Aspect	level	South	Southeast, Southwest	East, West, Flat terrain	Northeast, Northwest	North
4	Profile Curvature	radians/m	3.41–2.97	1.61–2.97	0.25–1.61	−1.10–0.25	−2.99–−1.1
5	Ruggedness Index	level	0–80	80–116	116–161	161–239	239–422.25
6	Lineament density	km/km^2^	0.33–0.41	0.24–0.33	0.16–0.24	0.08–0.16	0–0.08
7	TPI	level	−17.88–−2.55	−2.54–−0.81	−0.8–0.63	0.64–2.51	2.52–19
8	Lithology	level	Sand, Silt and Clay, Yellowish Brown Fine Sand, Silt, Clay, Gravel Beds, Volcanic Agglomerates, Sand, Silt, Clay, Calcareous Concrete	Lateritic Soil, Laterite, Limestone, Impure Marble, Calc Silicate Rocks, Limestone, Grit, Arkose, Conglomerate, Calc Schist, Dolomite	Chlorite-Sericite Schist, Mica Schist, Quartzite, Cherty Quartzite, Coarse Tuff, Quartz Vein, Reef, Hematite, Magnetite Quartzite, Hematite	Metabasic Rocks, Chlorite Schist And Altered Lava, Pelitic Schist, Granite Gneiss, Epidiorite, Hornblende Schist, Mafic Intrusive	Slate, Phyllite, Mica Schist, Gabbro-Anorthosite, Slate And Phyllite, Gabbro, Diorite, Staurolite Kyanite Schist, Phyllite, Shale
9	Geomorphology	level	Older Alluvial Plain, Older Flood Plain, Active Flood Plain, River, Dam and Reservoir, Pond, Waterbodies - unclassified	Low Dissected Structural Hills and Valleys, Moderately Dissected Structural Hills and Valleys, Highly Dissected Structural Hills	Highly Dissected Denudational Hills and Valleys, Moderately Dissected Denudational Hills and Valleys, Low Dissected Denudational Hills	Highly Dissected Structural Upper Plateau, Highly Dissected Structural Lower Plateau, Moderately Dissected Structural Upper Plateau	Active Quarry
(B) Hydrologic							
10	TWI	level	15.67–24.73	11.66–15.67	8.73–11.66	6.45–8.73	0–6.45
11	SPI	level	0–5.0	5.01–20.16	20.17–42.84	42.83–76.23	>76.23
12	Drainage density	km/km^2^	0.9–2.2	2.3–2.9	2.91–3.54	3.541–3.98	>3.98
13	Precipitation	mm	1663–1753	1565–1663	1465–1563	1385–1465	1220–1385
14	Distance from rivers	m	<500	500–1000	1000–1500	1500–2000	>2000
15	Total RO	kg m^−2^ s^−1^	0.022–0.037	0.037–0.058	0.058–0.083	0.083–0.104	0.104–0.141
16	Baseline water stress	%	<10	10–20	20–30	30–40	>80
(C) Soil permeability							
17	Soil type	level	Sandy clay Loam	Loam	Sandy Loam	Clay Loam	Clay
18	Soil moisture	mm	17.70–21.97	13.43–17.70	9.17–13.43	4.90–9.17	0.63–4.90
19	Soil ET	kgm^−2^ s ^−1^	0.000045–0.000049	0.000042–0.000045	0.000040–0.000042	0.000038–0.000040	0.000034–0.000038
20	Soil loss	t ha^−1^ yr^−1^	0.08–0.56	0.56–1.1	1.1–1.8	1.8–3.5	3.5–6.1
(D) Terrain distribution							
21	LU/LC dynamics	level	Water bodies, Wetland	Forest cover area, Agricultural land	Shrubland, Herbaceous vegetation	Bare land/Sparse vegetation	Settlement areas
22	NDVI	level	0.36–0.63, −0.02 - 0.015	0.27–0.36	0.18–0.27	0.14–0.18	0.015–0.14
23	GHMTS	level	0.14–0.40	0.40–0.50	0.50–0.60	0.60–0.70	0.70–1.0
(E) Anthropogenic							
24	Population density	Person/km^2^	<250	250–500	500–1500	1500–2500	>2500
25	HBSE	level	200	200–200.05	200.05–200.07	200.07–201	201–202
26	GMIS	%	175–200	150–175	125–150	100–125	0–100
27	Distance from road network	m	>2000	1500–2000	1000–1500	500–1000	<500

All conditioning factors are reclassified into raster layers with a resampled 30-m spatial resolution. Ten experts in the fields of hydrogeology, meteorology, and local administrators were interviewed through a questionnaire. Additionally, water resources experts were consulted to gather their opinions on these factors' relative importance and ranking. Furthermore, the weights of the factors were estimated using FuzzyAHP, FuzzyDEMATEL, and Logistic regression techniques. Considering the probability of influencing GWP, individual factors were classified into five levels of impact, including very high, high, moderate, low, and very low. The groundwater potentiality of individual conditioning factors was evaluated, and the combined groundwater potentiality level for the main factors was estimated. The ranges of the selected factors were classified into two categories: numerical and non-numerical. The factors with numerical ranges were closely observed to decide whether they influenced the GWPZ directly or inversely. The numerical ranges of the potential classes were divided through Jank's natural break grouping based on the available data range in ArcGIS software [[56], [57]].

### 2.6 Multi-collinearity analysis

Multi-collinearity denotes the linear dependency between two or more variables within a dataset [58]. As noted by Singha et al. [9], the Variance Inflation Factor (VIF) and tolerance levels, generated through multi-collinearity analysis, are valuable tools for GWPZ mapping (Eq. (1) & Eq. (2)).

(1) $$Toleranc=R_{J}^{2}$$

(2) $$VIF=\lfloor \frac{1}{Tolerance}\rfloor$$

where, $R_{J}^{2}$ is the coefficient of determination of J ^th^ factor. The threshold value of tolerance and VIF is > 0.1 and < 10, respectively [59]. The multicollinearity may exist when the value VIF exceeds 5.0, though a significant impact will be observed when it crosses 10.

### 2.7 Random forest (RF) modeling

RF modeling has been widely employed for nonparametric multivariate classification and regression tasks. RF works by constructing many decision trees during training and aggregating their outputs to improve predictive accuracy and control overfitting, making it a robust choice for complex datasets [60]. Our study employed the 'caret' package in R version 4.0.2 to implement the RF model. Specifically, after conducting multiple experiments, we configured the number of trees ('ntree') to 500 and set the 'mtry' parameter to 10. We adopted a 10-fold cross-validation approach during the RF modeling process to enhance the model's robustness and mitigate overfitting. The GWCFs were prioritized based on the mean decrease in accuracy using the Gini index. We ensured that the total number of trees and factors tested at each split were fixed at 500 and 5, respectively, to achieve the lowest Out-of-bag (OOB) error (Supplementary Fig. 2).

### 2.8 MCDM modeling

In the FAHP modeling, all parameter weights are established relying on the decisions made by the decision-maker and their preferences for the alternatives. The analysis was conducted in the Python Google Colab cloud environment to generate the final results.

### 2.9 Fuzzy-AHP modeling

Fuzzy AHP represents an updated version of the AHP method introduced by Saaty. Given the inherent uncertainty and vagueness associated with the AHP method, FAHP is considered a more effective alternative. The fuzzy system incorporates fuzzification, which involves converting linguistic terms into membership functions. These membership functions consist of three parts: lower, upper, and intermediate membership functions. In our study, the triangular fuzzy number (TFN) matrix is implemented to manage uncertainty and ambiguity through the membership system (Supplementary Fig. 3). TFNs are typically denoted as (l, m, u) or (l/m, m/u), representing the lowest possible value, the highest possible value, and the most likely value, respectively. The scale of relative importance, which ranges from 1 to 9 in crisp numbers, is replaced with fuzzy numbers (FNs). Each term is assigned a value between 0 and 1, representing its degree of membership within the intersection numbers. TFNs have a linear representation of each degree of membership on their left and right sides, as described in (Eq. (3) & Eq. (4)) [61].

(3) $$\mu \left(x\setminus \tilde{M}\right)=\left\{\begin{array}{cc} 0\text{,} & x<l\text{,}\\ \left(x-l\right)/\left(m-l\right)\text{,} & l\leq x\leq m\\ \left(u-x\right)/\left(u-m\right)\text{,} & m\leq x\leq u\\ 0\text{,} & x>u \end{array}\right\}$$

(4) $$\begin{array}{c} \tilde{M}=\left(M^{l\left(y\right)},M^{r\left(y\right)}\right)\\ \left(l+m-l\right)y,u+\left(m-u\right)y) \end{array}$$

where, l(y) and r(y) denote the left and right sides of a FNs, respectively.

To determine the weights of evaluation criteria using FAHP, pairwise comparison matrices were formulated for all criteria within the hierarchy which is expressed as matrix $\tilde{A}$ in (Eq. (5)):

(5) $$\tilde{A}=\left[\begin{array}{cccc} \tilde{1} & {\tilde{a}}_{12} & \ldots & {\tilde{a}}_{1n}\\ {\tilde{a}}_{21} & \tilde{1} & & {\tilde{a}}_{2n}\\ \vdots & \vdots & \ddots & \vdots \\ {\tilde{a}}_{n1} & a_{n2}\cdots & \tilde{1} & \end{array}\right]=\left[\begin{array}{cccc} \tilde{1} & {\tilde{a}}_{12} & \ldots & {\tilde{a}}_{1n}\\ 1/{\tilde{a}}_{21} & \tilde{1} & & {\tilde{a}}_{2n}\\ \vdots & \vdots & \vdots & \vdots \\ 1/{\tilde{a}}_{n1} & 1/a_{n2}\cdots & \tilde{1} & \end{array}\right]$$

where, ${\tilde{a}}_{ij}$ the relationship between parameters i and j. When parameters i and j are identical (i.e., i=j), the notation $\tilde{1}$ is defined as a triangular fuzzy number represented by (1), (1), (1). Extending this concept, a fuzzy scale ranging from $\tilde{1}$ to $\tilde{9}$ is employed to assess the relative importance of parameters i to j. Conversely, the inverse of this scale, ranging from ${\tilde{1}}^{-1}$ to ${\tilde{9}}^{-1}$, is utilized to measure the relative importance of parameters i to j. This nuanced approach in evaluating the significance of parameters in relation to each other is underpinned by a fuzzy transformation rating scale, the specifics of which are elucidated by Tahria et al. [62].

Subsequently, the fuzzified pairwise comparison matrix was estimated using the Buckley fuzzy system to calculate the final fuzzy weighting through the geometric mean method (Eq. (6)).

(6) $$\begin{array}{c} {\tilde{r}}_{i}={\left({\tilde{a}}_{i1}⊗{\tilde{a}}_{i2}⊗\ldots ⊗{\tilde{a}}_{in}\right)}^{1/n}\\ \;\text{And},\text{then}\;{\tilde{w}}_{i}={\tilde{r}}_{i}⊗{\left({\tilde{r}}_{i}⊗\ldots ⊗{\tilde{r}}_{n}\right)}^{-1} \end{array}$$

where, ${\tilde{a}}_{in}$ is fuzzy comparison value of criterion i to criterion n; therefore, ${\tilde{r}}_{i}$ is the geometric mean of fuzzy comparison value of criterion i to each criterion. In ${\tilde{w}}_{i}$ , *i* is the fuzzy weight of the i th criterion, and it can be revealed by a TFN. ${\tilde{w}}_{i}=\left({lw}_{i},uw_{i},mw_{i}\right)$, where ${lw}_{i}$*,* $uw_{i}$, $mw_{i}$*,* stand for the lower, upper, and intermediate values of the fuzzy weight of the i th criterion, respectively.

In this research, the principle of linguistic pairwise comparison was employed to estimate hierarchical fuzzy weights. Fuzzy weights were defined for the five selected GWPZ main criteria using fuzzy numbers, and these weights were further processed through the center of the area as a defuzzification method. This method calculated the defuzzified numeric crisp weights, which were then used to estimate the normalized final weight of the GWP influencing factor.

### 2.10 Fuzzy DEMATEL modeling

DEMATEL is a group decision-making tool used to address complex inter-criterion relationship problems by interpreting causal effects [63]. Expert opinions, judgments, and respondent views are assigned numerical scores on a scale of five levels (0–4) for each perception. To account for inconsistencies and vagueness in expert opinions and subjectivity, we computed the initial direct influence matrix by converting the linguistic variables into corresponding TFNs (Triangular Fuzzy Numbers) (Eq. (7)), as shown in Supplementary Table 1.

(7) $$\left[\begin{array}{cccc} 0 & x_{12} & \ldots & x_{1n}\\ x_{21} & 0 & \ldots & x_{2n}\\ \vdots & \vdots & \vdots & \vdots \\ x_{n1} & x_{n2} & \ldots & 0 \end{array}\right]$$

The initial direct influence matrix is built through several pair-wise comparison matrix. Computed the initial fuzzy direct-relation matrix $Z^{k}$ by having inspectors acquaint with the fuzzy pair-wise relation between the entities in an n×n matrix, where *k* is the number of respondents. Based on the direct-relation, matrix is recognized as $Z^{k}$ = [$Z_{ij}^{k}$]; Where Z is n×n non-negative matrix; $Z_{ij}$ denotes the direct influence of factor i on factor j; and, when i = j and the diagonal features $Z_{ij}=0$ (Eq. (8)).

(8) $$\begin{array}{c} z_{ij}^{k}=\left(l_{ij},m_{ij},u_{ij}\right)\\ C_{1}\\ Z^{k}=\left[\begin{array}{ccccc} \left[0,0\right] & ⊗z_{12}^{k} & \ldots & ⊗z_{1n}^{k} & \\ {⊗}_{2} & ⊗z_{21}^{k} & \left[0,0\right] & \ldots & ⊗z_{2n}^{k}\\ \vdots & \vdots & \vdots & \vdots & \\ C_{n} & ⊗z_{n1}^{k} & ⊗z_{n2}^{k} & \ldots & \left[0,0\right] \end{array}\right] \end{array}$$

Calculated the normalized fuzzy direct-relation matrix “D” using the following equation (Eq. (9))

(9) $$D=\frac{Z^{k}}{\max_{1\leq i\leq n}\;\sum_{j=1}^{n}\;z_{ij}},i,j=1,2,\ldots ,n$$

Obtained the total-relation matrix T using Eq. (8), where n × n identity matrix is denoted with I. Lower and upper values are computed individually from the formula (Eq. (10)).

$$T=D{\left(I-D\right)}^{-1}$$

where,

(10) $$T=D+D^{2}+\ldots +=\sum_{i=1}^{\infty }\;D^{i}$$

Acquiring the column (*C*_*j*_) and row (R_i_) sums for each column j and row i from the T matrix, respectively, using equation (Eq. (11)).

(11) $$\begin{array}{cc} T={\left[t_{ij}\right]}_{nxn} & i,j=1,2,\ldots n\\ R_{i}=\sum_{1\leq j\leq n}^{n}\;t_{ij} & \forall i\\ C_{j}=\sum_{1\leq i\leq n}^{n}\;t_{ij} & \forall j \end{array}$$

The final Fuzzy DEMATEL weight $W_{j}$ of criteria acquired from the equation (Eq. 12)

(12) $$W_{j}=\frac{\sum_{j=1}^{n}\;\left(R_{i}-C_{i}\right)}{\sum_{i=1}^{n}\;\sum_{j=1}^{n}\;\left(R_{i}-C_{i}\right)}$$

The criterion cause and effect prominence/relation is shown by the causal diagram, where horizontal axis is represented by $\left(R_{i}+C_{i}\right)$ and the vertical axis is defined by $\left(R_{i}-C_{i}\right)$. The “Prominence” indicator was demarcated in the horizontal axis, represented the relative importance of each parameter. The “Relation” is indicated in the vertical axis with the extent of influence of the factor. In this intersection module, the cause group is exhibited when the $\left(R_{i}-C_{i}\right)=>0$, otherwise $\left(R_{i}-C_{i}\right)=<\;0$ condition is contained in the effect group of the factor. Causal graph delineating the two-dimensional space for judgment of the multi-decision support system environment identifying the most influential factor and how they are interdependent with other parameters. In this study, Fuzzy DEMATEL methods were applied using Python jupyter notebook inbuilt GoogleColab cloud environment.

### 2.11 Logistic regression modeling

The logistic regression model elucidates the GWPZ probability association between two intervariables [64]. The model output could be binary components, such as 0 or 1, high (100 % potential) or low (0 % potential) on a sigmoid-shaped curve. Logistic function p(z) working in this model is defined by the following formula (Eq. 13)

(13) $$p\left(z\right)=\frac{1}{1+e^{-z}}$$

where, z is the net input of linear combination which is the linear combination of weights (β) and sample features (x), and given by the following equation (Eq. (14)). The probability of the best-fitting model was derived from logit transformation (Eq. (15)).

(14) $$z=\beta^{T}x=\beta_{0}+\beta_{1}x_{1}+\beta_{2}x_{2}+\ldots +\beta_{n}x_{n}$$

(15) $$\text{logit}=\log\;\left\{\frac{p\left(x\right)}{1-p\left(x\right)}\right\}$$

where the coefficients β are computed using the maximum likelihood function. ($\beta_{0}$ represents the intercept of the model, and $\beta_{i}$ represents the criteria. p(x) denotes the odds ratio that explained the occurrence of the potential probability.

## 3 Results

### 3.1 Multi-collinearity application

In this section, we present the results of our analysis on multicollinearity among twenty-seven influential parameters categorized into five major criteria for GWPZs. We examine the tolerance and VIF values to assess multicollinearity issues affecting the GWCFs. Tolerance and VIF values below 0.1 and less than 10, respectively, indicated the absence of significant multicollinearity issues among predictor variables. The multicollinearity test result (Table 3) demonstrated no multicollinearity problems affecting the effectiveness of groundwater potential, as all the GWCFs had tolerance and VIF values below the specified thresholds. Tolerance values for the factors ranged from 0.13 to 0.85, while VIF values ranged from 1.16 to 7.69. Aspect had the highest tolerance value (0.85) among the conditioning factors, while HBSE had the highest VIF value (7.69).

**Table 3:** Results of Multi-collinearity analysis among the GWCFs.

Criterion	Tolerance	VIF
Elevation	0.41	2.44
Slope	0.21	4.84
Aspect	0.86	1.17
Profile Curvature	0.28	3.56
RI	0.61	1.64
TPI	0.24	4.10
Lineament	0.65	1.55
Geomorphology	0.78	1.28
Lithology	0.45	2.21
CHIRPS	0.23	4.36
SPI	0.14	7.07
TWI	0.14	7.41
DD	0.59	1.71
Dist.to river	0.67	1.50
TRO	0.32	3.12
ST	0.34	2.91
SM	0.44	2.29
SL	0.28	3.54
ET	0.26	3.86
NDVI	0.57	1.75
LULC	0.36	2.76
GHMTS	0.48	2.08
PD	0.63	1.60
HBSE	0.13	7.69
GMIS	0.14	7.46
Dist.to road	0.72	1.39

### 3.2 RF application for the relative criterion importance

The RF algorithm used 100 trees and 27 conditioning factors for the GWPZ estimation. It can be seen from Table 4 that the RF model correctly identified 34 non-potential wells as non-potential wells and 5 non-potential incorrectly as potential wells. Conversely, the algorithm correctly identified 28 groundwater potential wells as potential wells and 5 groundwater wells incorrectly as non-potential wells. Furthermore, the OOB error rate, estimated at 13.89 %, indicates an accuracy of 86.11 % in GWPZ estimation. The reduction in OOB error over the splits and tree numbers were depicted in Supplementary Fig. 2.

**Table 4:** The confusion matrix of RF model.

Class	non-potential (“0”)	potential (“1”)	Error
non-potential (“0”)	34	5	0.128
potential (“1”)	5	28	0.151

**Fig. 3:**
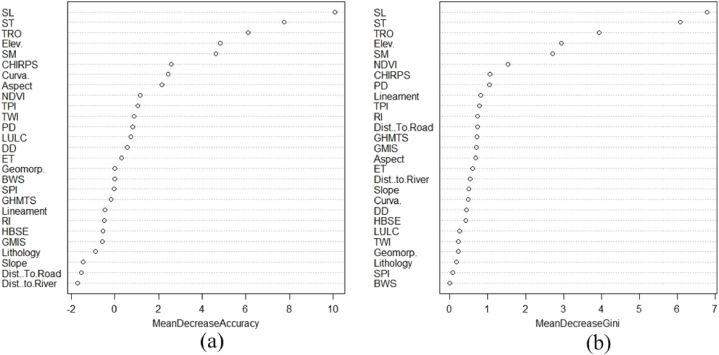
Variable importance analysis of (a) mean decrease accuracy and (b) mean decrease Gini for GWPZ influencing factors using the RF model.

The relative importance of the GWCFs derived from RF model in Fig. 3 (a) and (b) revealed that soil loss (6.78), soil type (6.08), total runoff (3.94), elevation (2.94), and soil moisture (2.71) were the most critical sub-criteria in the GWPZ analysis in the study area, while SPI (0.08) and baseline water stress (0.001) were the minor important criteria (Table 5).

**Table 5:** Assessing the relative significance of the GWCFs.

Sl. No.	Criterion	0	1	Mean Decrease Accuracy	Mean Decrease Gini
1	Elev.	4.04	4.34	4.84	2.94
2	Slope	−0.94	−1.19	−1.45	0.50
3	Aspect	2.32	0.92	2.16	0.69
4	Curva.	2.43	0.39	2.44	0.49
5	RI	−0.12	−0.53	−0.51	0.73
6	TPI	1.51	−0.06	1.05	0.78
7	Lineament	−0.41	−0.32	−0.46	0.82
8	Geomorp.	−1.01	1.01	0.00	0.23
9	Lithology	−0.63	−1.01	−0.90	0.18
10	CHIRPS	1.23	3.24	2.57	1.06
11	SPI	0.58	−0.79	−0.03	0.08
12	TWI	1.01	−0.04	0.88	0.23
13	DD	1.33	−1.13	0.56	0.44
14	Dist. to.River	0.05	−1.71	−1.71	0.53
15	BWS	0.001	0.001	0.001	0.001
16	TRO	5.30	5.01	6.10	3.94
17	ST	7.00	5.81	7.75	6.08
18	SM	3.86	4.01	4.63	2.71
19	SL	9.63	6.97	10.08	6.78
20	ET	1.19	−0.60	0.32	0.61
21	NDVI	0.24	1.41	1.14	1.53
22	LULC	−0.47	1.53	0.74	0.26
23	GHMTS	0.38	−0.68	−0.17	0.72
24	PD	−0.53	1.52	0.82	1.05
25	HBSE	0.001	−0.63	−0.54	0.43
26	GMIS	−1.42	0.06	−0.58	0.70
27	Dist.To.Road	−2.08	0.17	−1.54	0.72

### 3.3 GWPZs with main criteria

**Fig. 4:**
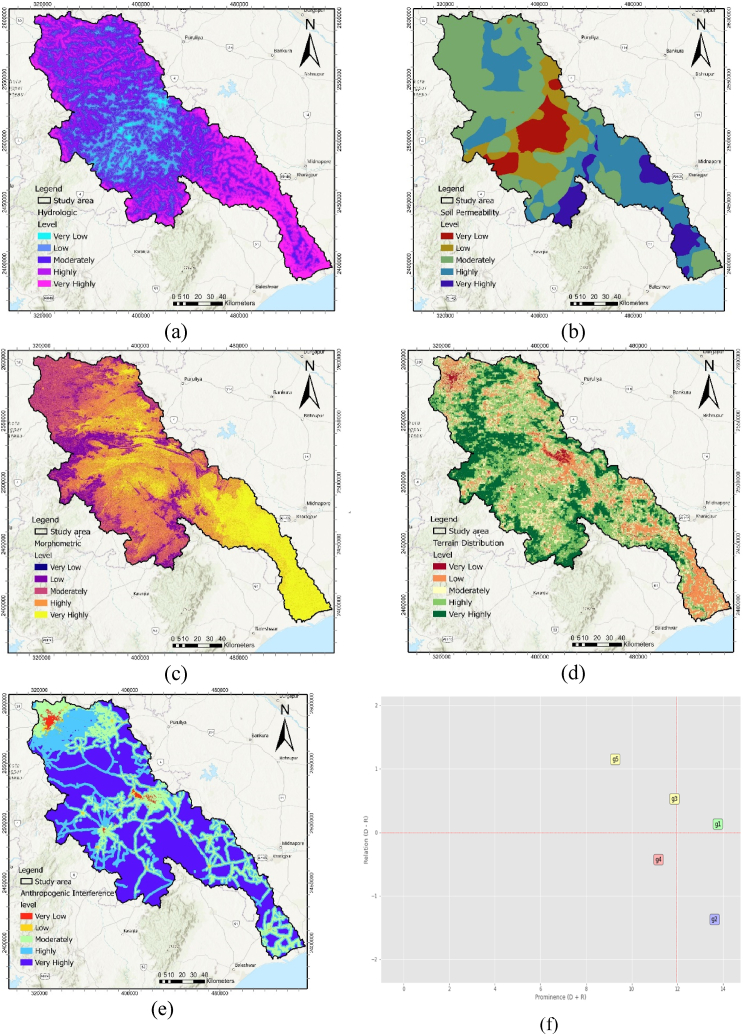
GWPZ main criteria distribution map: (a) Hydrologic, (b) Soil permeability, (c) Morphometric. (d) Terrain distribution, (e) Anthropogenic, and f) Casual graph for g1∼hydrologic, g2∼soil permeability, g3∼morphometric, g4∼terrain distribution, g5∼anthropogenic criteria.

We employed 27 sub-factors, categorized into five major groups, to predict groundwater potential. The suitability of each parameter was assessed within these five significant criteria. A groundwater potential map was generated by integrating sub-criteria with individually reclassified layers using the natural break (Jenks) method, resulting in five-level classifications. These five central criteria-based GWPZ maps were then used to generate various output maps through MCDM models. In terms of hydrologic criteria, the highly and very highly potentiality classes covered 23.86 % and 18.73 % of the total area, respectively (Table 6 & Fig. 4 (a)). Permeability and terrain distribution were the most influential criteria for very low potentiality, with shares of 8.73 % and 1.46 %, respectively (Fig. 4 (b) and (d)). Regarding morphometric criteria, the moderately potential class accounted for 26.71 %, while the highly potentiality class covered 34.52 % (Fig. 4 (c)). In the anthropogenic criterion, areas with low to very low potentiality constituted 0.91 %. In comparison, 19.01 % of the areas fell under the moderately potential class (Fig. 4 (e)). The highest GWP class, covering 50.11 % of the area, was associated with the very highly potential category for the anthropogenic criterion (Table 6).

**Table 6:** Area coverage of GWPZ classes for five main criteria.

Criteria	Very Low (sq. km)	%	Low (sq. km)	%	Moderately (sq. km)	%	Highly (sq. km)	%	Very highly (sq. km)	%
Morphometric	1.51	0.01	2234.0	11.53	5176.00	26.71	6689.00	34.52	5275.0	27.23
Hydrologic	620.86	3.20	3439.25	17.75	7062.79	36.45	4623.74	23.86	3628.88	18.73
Soil permeability	1691.11	8.73	2079.09	10.73	8521.34	43.98	5530.96	28.55	1553.02	8.02
Terrain distribution	282.05	1.46	3728.44	19.24	3982.38	20.55	6817.54	35.19	4565.10	23.56
Anthropogenic	176.41	0.91	177.19	0.91	3682.80	19.01	5629.60	29.06	9709.51	50.11

### 3.4 MCDM model derived GWPZs map

The MCDM models generated a GWPZ map for the Subarnarekha basin, classifying it into five primary levels. Fuzzy AHP, Fuzzy DEMATEL, and the Logistic Regression model were analyzed using an open-source Python Jupyter Notebook hosted on the Google Colab cloud platform.

#### 3.4.1 Fuzzy AHP estimation

**Fig. 5:**
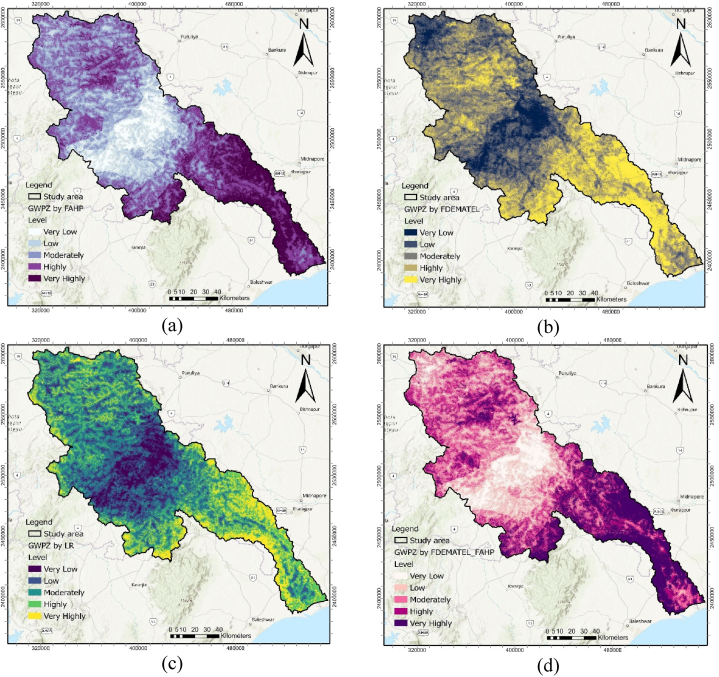

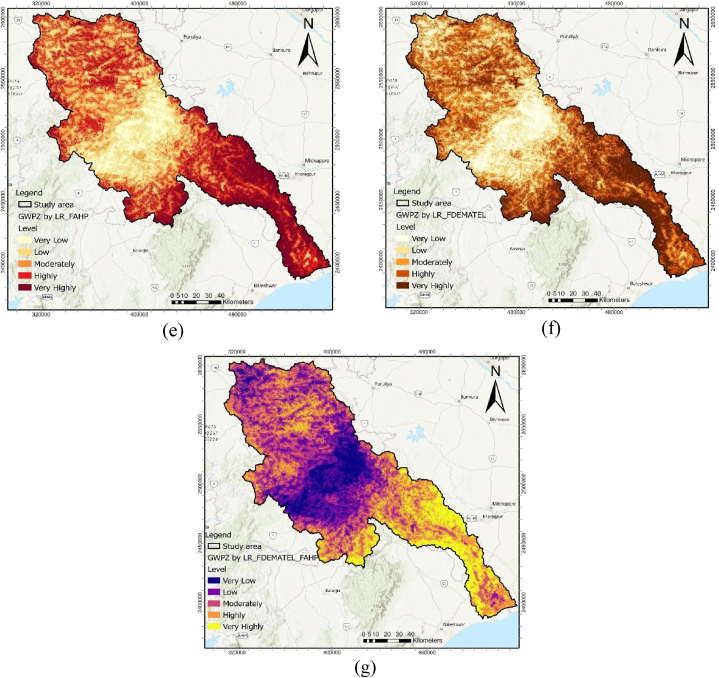
Final MCDM derived GWPZ map; (a) FAHP, (b) FDEMATEL, and (c) LR (d) FAHP-FDEMATEL, (e) FAHP-LR, (f) FDEMATEL-LR, and (g) FAHP-FDEMATEL-LR.

The Fuzzy AHP method was used to estimate the individual criterion weights, which were then employed to create the GWPZ map for the Subarnarekha basin. The fuzzy membership function was utilized to normalize the final fuzzy weighted criteria derived from fuzzy crisp layers (Table 7). Among the five criteria, the most influential factor in mapping the GWPZ was the hydrologic factor, with a weight of 0.410; thus, it was assigned the highest rank, indicating its suitability for groundwater recharge conditions. Conversely, the soil permeability, morphometric, terrain distribution, and anthropogenic parameters were measured less significant, with weights of 0.314, 0.162, 0.070, and 0.044, respectively. The groundwater potentiality zones were categorized into five classes: very highly, highly, moderately, low, and very low, covering areas of 3235.58 km^2^ (16.70 %), 5198.50 km^2^ (26.83 %), 5421.03 km^2^ (27.98 %), 3705.35 km^2^ (19.1 %), and 1815.04 km^2^ (9.37 %), respectively. The areas with very high and high groundwater potential were concentrated in the lower part of the Subarnarekha River (Fig. 5 (a)). In contrast, areas with moderate and low GWPZs dominated the upper and middle parts of the catchment area.

**Table 7:** Fuzzy AHP analysis for the assessment of the criterion weight and rank.

Criterion	Hydrologic	Soil Permeability	Morphometric	Terrain Distribution	Anthropogenic	Crisp weight	Normalized weight	Rank
Hydrologic	(1, 1, 1)	(1, 2, 3)	(2, 3, 4)	(4, 5, 6)	(6, 7, 8)	0.445	0.410	1
Soil Permeability	(1/3, 1/2, 1)	(1, 1, 1)	(2, 3, 4)	(4, 5, 6)	(5, 6, 7)	0.34	0.314	2
Morphometric	(1/4, 1/3, 1/2)	(1/4, 1/3, 1/2)	(1, 1, 1),	(2, 3, 4)	(4, 5, 6)	0.176	0.162	3
Terrain Distribution	(1/6, 1/5, 1/4),	(1/6, 1/5, 1/4),	(1/4, 1/3, 1/2),	(1, 1, 1)	(1, 2, 3)	0.076	0.070	4
Anthropogenic	(1/8, 1/7, 1/6),	(1/7, 1/6, 1/5),	(1/6, 1/5, 1/4),	(1/3, 1/2, 1)	(1, 1, 1)	0.048	0.044	5

#### 3.4.2 Fuzzy DEMATEL estimation

The study integrates fuzzy sets and the DEMATEL system to develop a comprehensive framework for measuring groundwater potential in the Subarnarekha catchment area. The interplay between each criterion was determined using linguistic variables and triangular fuzzy numbers (TFN) (Table 8). The study results were elucidated through causal graph analysis. In this analysis, groundwater potential GWP condition criteria such as g1 (hydrologic, 0.129), g3 (morphometric, 0.526), and g5 (anthropogenic, 0.155) were categorized into the cause criteria cluster. Meanwhile, the effect criteria cluster included g2 (soil permeability, −1.376) and g4 (terrain distribution, −0.431) in GWPZ modeling (Fig. 4 (f)). The cause cluster criteria depicted the impact of the influencing factors, while the effect cluster criteria referred to the consequences of the affected criteria. Soil permeability emerged as the most important criterion in GWP, with the highest Di-rj value of −1.376 (Table 8). In contrast, anthropogenic factors, with a higher Di + rj value of 1.152, were identified as significant criteria for GWPZ. Di + rj, termed as "prominence," was used to determine which parameters had the most substantial overall influence in the system. The results indicated that hydrologic criteria exhibited the most robust interactions with other parameters.

**Table 8:** Fuzzy DEMATEL analysis for the assessment of the criterion crisp, weight, and rank.

Criterion	Hydrologic	Soil permeability	Morphometric	Terrain distribution	Anthropogenic	Di + rj	Di-rj	Normalized Weight	Rank
Hydrologic	(0, 0, 1/4)	(3/4, 1, 1)	(1/2, 3/4, 1)	(1/4, 1/2, 3/4)	(0, 1/4, 1/2)	13.773	0.129	0.231	1
Soil permeability	(1/2, 3/4, 1)	(0, 0, 1/4)	(1/4, 1/2, 3/4)	(1/4, 1/2, 3/4)	(0, 1/4, 1/2)	13.632	−1.376	0.228	2
Morphometric	(1/2, 3/4, 1)	(1/2, 3/4, 1),	(0, 0, 1/4)	(0, 1/4, 1/2)	(0, 1/4, 1/2)	11.875	0.526	0.199	3
Terrain distribution	(1/4, 1/2, 3/4)	(1/4, 1/2, 3/4)	(0, 1/4, 1/2)	(0, 0, 1/4)	(1/4, 1/2, 3/4)	11.872	−0.43	0.187	4
Anthropogenic	(0, 1/4, 1/2)	(1/4, 1/2, 3/4)	(0, 1/4, 1/2)	(1/2, 3/4, 1)	(0, 0, 1/4)	9.281	1.152	0.155	5

Based on FDEMATEL, normalized weights for the respective conditioning criteria were utilized to generate the GWPZs map. Hydrologic criteria carried the highest weight (0.231) and ranked 1st, followed by soil permeability (0.228) in second rank, morphometric (0.199) in third rank, terrain distribution (0.187) in fourth rank, and anthropogenic (0.155) at fifth rank. Approximately 18.80 % of the study area was categorized as very highly groundwater potential zones (Fig. 5 (b)). Additionally, 28.45 % and 28.94 % were designated as highly and moderately potential areas, respectively. In comparison, 7.71 % of the area was classified as having very low groundwater potential zones (Table 9).

**Table 9:** MCDM model derived GWPZs area coverage in sq. km and percentage.

Model	Very Low		Low		Moderately		Highly		Very highly	
	(km^2^)	%	(km^2^)	%	(km^2^)	%	(km^2^)	%	(km^2^)	%
LR	1943.1	10	4553.9	23.5	5966.7	30.8	4361.4	22.5	2550.5	13.2
DEMA	1494.8	7.7	3257.8	16.8	5607.2	28.9	5513.1	28.5	3502.6	18.1
FAHP	1815	9.4	3705.4	19.1	5421	28	5198.5	26.8	3235.6	16.7
FDEM_FAHP	2202.1	11.4	4155.6	21.5	5676.7	29.3	3084.6	15.9	4256.5	22
LR_FAHP	2286.3	11.8	4468.5	23.1	3945.3	20.4	4852.9	25.1	3822.4	19.7
LR_DEMA	2251.1	11.6	4631.1	23.9	3021.7	15.6	5630.5	29.1	3841.1	19.8
LR_FDEM_FAHP	2530.5	13.1	4546.6	23.5	4456.2	23	4989.8	25.8	2852.5	14.7

#### 3.4.3 Logistic regression estimation

The LR model yielded coefficients (β) for independent criteria in the GWPZ map equation (Eq. (16)):

(16) Z=−13.59+0.464Xhydrologic+0.114Xsoilpermeability+0.019Xmorphometric+0.218Xterraindistribution−0.073Xanthropogenic

These conditional criteria were utilized to predict groundwater resources and estimate the GWPZ probability map. Positive coefficients (β) for hydrologic, soil permeability, morphometric, and terrain distribution positively influence groundwater occurrence probability. Conversely, the negative coefficient value (β) for anthropogenic (−0.073) diminishes the likelihood of groundwater occurrence, making it a less significant parameter. By examining the inter-relationships between dependent and independent criteria using the LR model, the GWPZ map was created (Fig. 5 (c)). It reveals varying groundwater potential levels, from very low (darker blue) to very high (lighter yellow) in the southeastern part. Finally, the GWPZ levels were reclassified into the following classes: very highly (13.16 %), highly (22.51 %), moderately (30.80 %), low (23.50 %), and very low (10.03 %) (Table 9).

#### 3.4.4 Hybrid MCDM modeling

An integrated MCDM modeling approach was used to create a GWPZ overlay layer in ArcGIS to minimize uncertainties in model performance. The combination of FAHP and FDEMATEL models resulted in a groundwater potentiality map (Fig. 5 (d)), with approximately 29.03 % indicating moderate potential (see Table 9).

#### 3.4.5 Integrated of FAHP and LR model

**Fig. 6:**
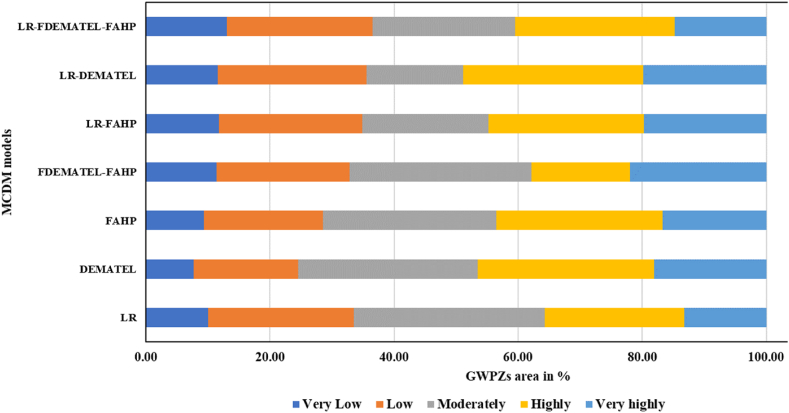
Area coverage of GWPZs in MCDM model.

Correspondingly, the combination of the FAHP and LR models revealed that within the total area of the Subarnarekha River basin, 19.73 % was categorized under the very highly GWP zone (Fig. 5 (e)). Additionally, 25.05 % and 20.36 % belonged to the highly and moderately potential classes, respectively. The spatial distribution of GWP results via the combined LR and FDEMATEL approach indicated that 48.88 % of the area had highly to very high GWPZ (Fig. 6). Furthermore, 15.6 % exhibited moderate potential, while low and very low potential areas covered 11.62 % and 23.9 % of the region, respectively (Fig. 5 (f)). In the final hybrid results of Fuzzy-AHP-DEMATEL-LR, very highly, highly, moderately, low, and very low GWP classes accounted for 14.7 % (2852.45 km^2^), 25.75 % (4989.78 km^2^), 23.0 % (4456.22 km^2^), 23.4 % (4546 km^2^), and 13.06 % (2530.48 km^2^) of the entire basin, respectively (Fig. 6). The model's output indicated that the downstream areas of the river basin exhibited high prospect zones due to their flat alluvial soil topography (Fig. 5 (g)). These regions have a high infiltration rate, resulting in greater aquifer storage capacity. In contrast, the recharge rate was identified as low in the northern and central parts of the basin, classifying them under the low and very low potential zone classes.

#### 3.4.6 Validation of groundwater levels

**Fig. 7:**
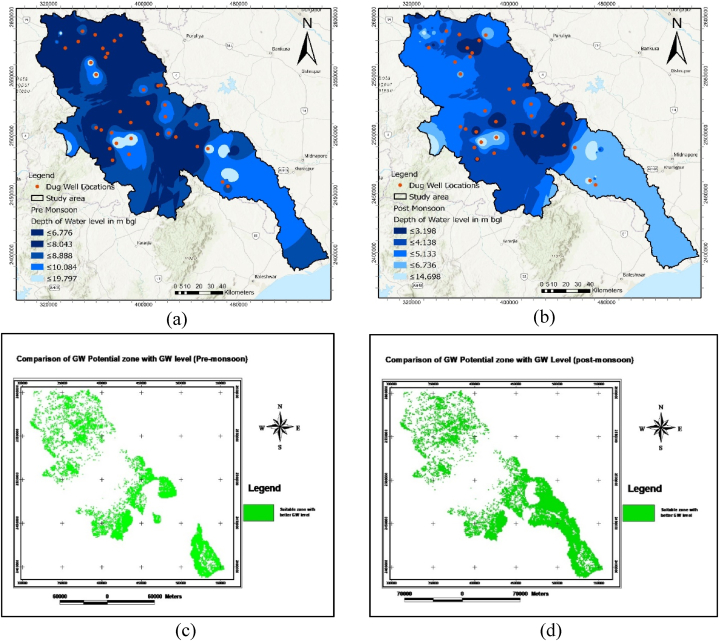
Comparison of GW depth in potential zones; a) Groundwater depth during pre-monsoon, (b) Groundwater depth during post-monsoon, (c) Suitable GW potential zones with better GW depth table: Pre-monsoon, d) Suitable GW potential zones with better GW depth table: Post-monsoon.

The GWPZ map was evaluated by long-term groundwater level data for pre- and post-monsoon periods. A groundwater depth map of the selected study area was created for both pre-and post-monsoon periods, categorized into five classes based on the water table depth of groundwater level (Fig. 7 (a) and (b)). The average groundwater level depth in the pre- and post-monsoon seasons varied from 4.35 to 7.57 m below groundwater level (mbgl), respectively. A high fluctuation level indicated low potential, while low fluctuation denoted high potential. The central part of the basin exhibited a high water table depth over both the pre-and post-monsoon seasons, indicating low GWPZ.

In contrast, the upper and lower parts of the study area were characterized as having high potential for groundwater storage throughout the entire basin which is align with the groundwater potential zone map developed through our model. The 'Map query' tool compared the groundwater potential zone map with the groundwater table depth. Most wells with low water table depths were located in the suitable GWPZ (very highly and highly classified) as mapped by the MCDM models for both the pre-monsoon and post-monsoon periods. Slightly reduced matching of both maps for pre-monsoon and post-monsoon GW depth may be attributed to the type of groundwater use in the south-central region of our study area (Fig. 7 (c) and (d)).

**Fig. 8:**
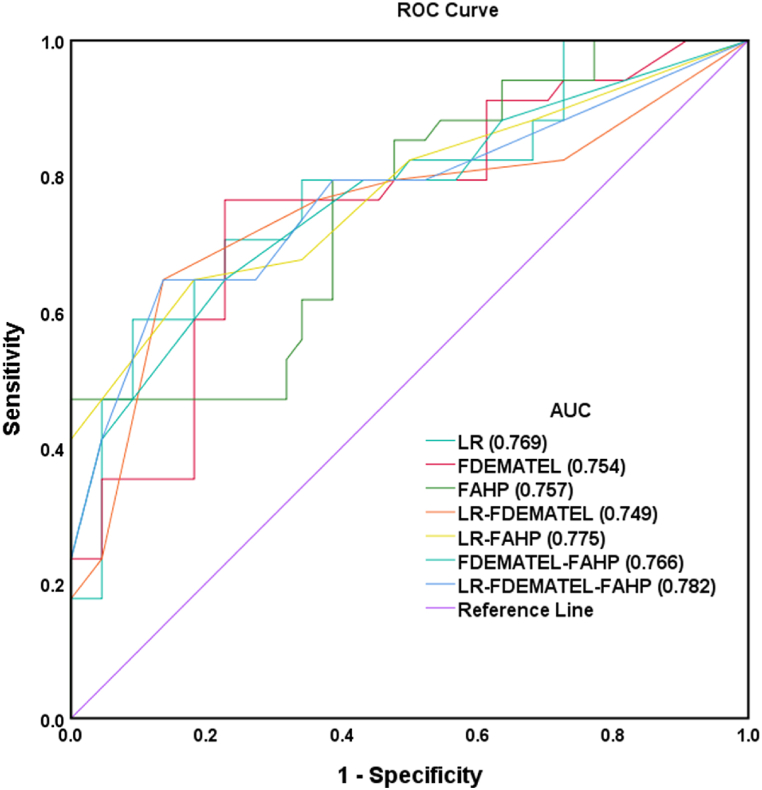
Validation of different MCDM models for GWPZs mapping using the AUROC curve.

The performance and efficiency of the Fuzzy-AHP, Fuzzy-DEMATEL, and LR models were evaluated using the AUC matrices. From the validation results, it can be concluded that the LR-Fuzzy-AHP-DEMATEL model demonstrated superior effectiveness in identifying groundwater potential areas. This model reflects the correlation between groundwater potential criteria and the assigned criterion weights obtained through an MCDM model. The AUC-ROC value of LR-FDEMATEL-FAHP (0.782) is higher than that of LR-FAHP (0.775). The AUC results indicate that LR-FAHP achieved approximately 78 %, LR achieved 77 %, and FDEMATEL-FAHP achieved 76 %. FAHP and FDEMATEL-LR both achieved approximately 75 % (Fig. 8). Therefore, the AUC results for the study area ranging between 75 % and 78 % indicate medium to the high predictability of GWPZ. An AUC value greater than 75 % is considered satisfactory for acceptable model performance in this context [65].

#### 3.4.7 Validation with actual pumping well yield

To certify the GWPZ map, yield data from pumping wells in the lower basin of the Subarnarekha watershed (Baliapal and Hasimpur blocks, Balasore district, Odisha, India) were utilized. In this region, the average well yield was approximately 2334.14 m³ per day, primarily used for irrigation. Nearly all existing pumping wells in the region fell within the "good" and "very good" categories as determined by the hybrid model. Out of the ten wells, nine yields agreed with the GWPZ mapping, and one partially agreed with the GWPZ map. Two wells, currently in a defunct condition, disagreed with the GWPZ map (Table 10). Therefore, the overall accuracy level of the hybrid MCDM model was approximately 77 %. While this accuracy level may not be very high compared to previous studies involving fewer factors, it is noteworthy that some studies utilizing machine-learning techniques have achieved similar levels of efficiency. For example, the AHP produced an accuracy of 78.8 % [66] with an AUROC score of 0.705, which was considered indicative of good prediction capability for AHP. Additionally, results using the LR algorithm on testing data showed an AUROC score of 0.686.

**Table 10:** Assessment of generated GWPZ map based on the well yield information.

Sl. No.	Well ID	Latitude	Longitude	Actual yield (m3/day)	Well location on GWP maps	Hybrid model
1	380	21.631	87.2562	2180.4	Very good	Agree
2	132	21.6563	87.2674	1817	Good	Agree
3	89	21.655	87.2951	1635.3	Good	Agree
4	44	21.6641	87.289	2725.5	Very good/good	Agree
5	5	21.67	87.2952	1998.7	Very good	Agree
6	138	21.67	87.2311	2725.5	Moderate	Defunct/Disagree
7	64	21.6715	87.2393	1726.15	Moderate	Defunct/Disagree
8	126	21.6747	87.2525	2089.55	Very good	Agree
9	52	21.6665	87.26	2543.8	Very good	Partially agree
10	216	21.7021	87.2004	1453.6	Very good	Agree
11	18	21.7027	87.2299	2271.25	Very good	Agree
12	130	21.6245	87.2856	5996.09	Good	Agree
13	33	21.6363	87.2717	1181.05	Good	Agree

## 4 Discussions

### 4.1 Groundwater status in the study area

In the study area, the continuous expansion of agricultural land, mining activities, deforestation, industrialization, and urbanization have led to an increasing demand for water, resulting in the depletion of the water table over time. Additionally, groundwater recharge decreases due to lower precipitation levels and intermittent rainfall events with limited runoff [67]. In such a scenario, there is a critical need for effective water management policies and training programs to enhance understanding of the hydrologic, morphometric, soil permeability, and anthropogenic factors interconnected with aquifer storage and surface runoff conditions. Consequently, groundwater resource mapping and analysis are crucial in monitoring long-term water sustainability and development within any region [23].

### 4.2 Selection of individual factors

Twenty-seven relevant individual factors were measured for mapping groundwater potential zones. With the availability of cloud computing platforms, the estimation of parameters for high-resolution and large-sized images can be carried out easily, quickly, and efficiently. Increasing the number of parameters can precisely identify groundwater monitoring parameters and provide accurate zone mapping. However, the proximity of some parameters may lead to overlapping influences. Therefore, similar properties and composition factors were grouped under the same primary criteria [54]. also employed this approach of grouping criteria for groundwater potential mapping in Pakistan. In addition, the weight allocated to each class in the different criteria thematic maps based on their water potential capacity and characteristics is determined using the AHP method [57]. The data on the region's groundwater prospects validated the method's accuracy.

In the MCDM modeling output, the hydrologic factor was the most crucial criterion, ranking highest in the GWPZ mapping analysis. The causal diagram revealed that the hydrologic (g1) criteria held the most significance, with high prominence and strong relationships compared to other criteria. Similarly, the terrain distribution (g4) criteria had less influence, with low prominence and weaker relationships than others. Morphometric (g3) and anthropogenic (g4) criteria were essential and could be influenced by other criteria, although they had low prominence and high relationships with other criteria. Lastly, the soil permeability criteria were essential and could not be significantly influenced by other criteria, with high prominence and low relationships.

### 4.3 Model integration as hybrid approach

To overcome the limitation of a single model and better represent all influencing factors, we integrated the MCDM models, namely, Fuzzy AHP [68] and Fuzzy DEMATEL, along with machine learning techniques, such as LR and RF, to achieve better groundwater zone mapping [69]. used logistic regression and RF to map groundwater at Sohag Governorate in Egypt [70]. created a GWPZ map in Upper Mesopotamia, Turkey, using ten theme layers through the Fuzzy AHP technique. All the different conditional criteria, modeling maps, calculations, and evaluations were conducted using fast-processing GIS, GEE, and the Google Colab cloud environment. The ROC validation results indicate that the hybrid (AUC∼78 %) model is a more effective MCDM-based tool for identifying GWPZ areas. This hybrid model reveals that very high to high potential GWP classes cover 40.45 % (7842.23 km2) of the entire basin.

### 4.4 Groundwater potential zoning

The results shown that high groundwater resources were situated in close vicinity to the river channel (<500 m), characterized by a gentle slope, high TWI, high soil moisture, and low drainage density (<2.2 sq km) around the riverbank area [71]. showed that slope, LULC, and soil factors were the most sensitive factors for assessing GWPZ in Bangladesh. The low and very low potentiality areas corresponded to high population density, built-up land, and a short distance to the road network. Low potentiality areas also featured low porosity lithological ingredients (such as slate, phyllite, mica schist, gabbro-anorthosite, slate, phyllite, gabbro, diorite, staurolite kyanite schist, phyllite, and shale) with low permeability, high runoff, steep slopes, high elevation, and significant soil erosion.

Implementing targeted measures for water conservation, such as groundwater recharge plans and various structural enhancements, is crucial. These actions, aligned with local terrain and geological features, are designed to bolster groundwater storage capacity in the study area.

The downstream and southern parts of the region exhibit good groundwater potential due to the presence of alluvial soil with a high infiltration rate, particularly in flat terrains. Generally, the southern regions experience higher groundwater recharge as runoff flows slower than the northern regions. Among the terrain distribution parameters, the NDVI indices serve as a reliable indicator of groundwater potential, particularly in the region's western part, where the NDVI value exceeds 0.55. The study's findings indicated that the NDVI could be utilized to identify areas with a shallow water table and natural vegetation and areas with inadequate in-situ observations [72]. In the land-atmospheric system, evapotranspiration and soil moisture strongly correlate with groundwater potential. In the lower stream of the entire study area, the presence of moderate to high evapotranspiration and soil moisture levels suggests the existence of an unconfined aquifer [73]. The Subarnarekha region faces significant soil loss due to extensive weathering and large-scale deforestation [12]. With a similar study, we could identify the susceptible zones and carry out necessary preventive measures in the river basin.

Moreover, baseline water stress rises due to illegal mining activities, agricultural water withdrawals, and industrial demands [11]. The RF modeling results highlight that soil loss and soil type are the most influential variables, contributing to an accuracy of 86.11 % in estimating groundwater potential in the study area. The RF model was found suitable for this type of study and could be extended to other regions for wider applicability of the model as demonstrated by Madani and Niyazi, [74] in Western Saudi Arabia.

### 4.5 Bourta Senstivity analysis

**Fig. 9:**
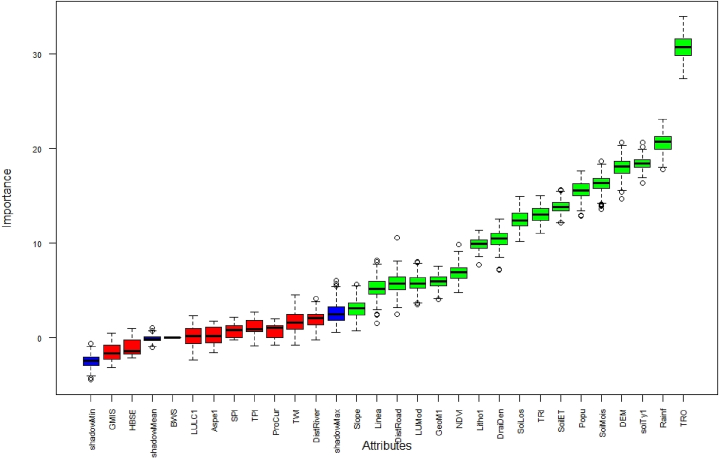
Boruta feature selection analysis for GWPZ factors.

Multi-collinearity-based critical analysis was carried out on the factors influencing final outputs. The selected parameters were evaluated through Boruta feature selection analysis to determine their level of significance (Fig. 9) GW distribution in the region. The least essential parameters could be included in the final potential zoning map [75]. The Boruta sensitivity of the groundwater influencing factors was summarized (Table 11). Based on the mean importance for the availability of the groundwater influencing factors, namely Total Runoff (30.74), rainfall (20.63), soil type (18.48), and DEM (18.02) are the most important factors, followed by soil moisture (16.31), population density (15.57), soil ET (13.85), TRI (13.02), soil loss (12.50), drainage density (10.47), lithology (9.90), NDVI (6.89), geomorphology (5.98), land use modification (5.81), road distance (5.77), lineament density (5.22), and slope (3.05). However, distance to rivers, BWS, TWI, TPI, SPI, profile curvature, aspect, LULC, HBSE, and GMIS were revealed to have been rejected among all assigned factors (Fig. 9). This will optimize the time required for analysis as well as improve the overall model accuracy.

**Table 11:** Analysis of GWPZ importance factor using by Boruta technique.

Parameters	meanImp	medianImp	minImp	maxImp	normHits	decision
TRO	30.74	30.77	27.41	34.03	1.00	Confirmed
Rainfall	20.63	20.76	17.79	23.11	1.00	Confirmed
soiType	18.48	18.47	16.36	20.66	1.00	Confirmed
DEM	18.02	18.10	14.72	20.69	1.00	Confirmed
SoiMoisture	16.31	16.34	13.59	18.69	1.00	Confirmed
Population	15.57	15.58	12.89	17.67	1.00	Confirmed
SoilET	13.85	13.82	12.15	15.70	1.00	Confirmed
TRI	13.02	13.04	11.07	15.01	1.00	Confirmed
SoilLoss	12.50	12.38	10.21	14.94	1.00	Confirmed
DrainageDensity	10.47	10.51	7.15	12.59	1.00	Confirmed
Lithology	9.90	9.94	7.71	11.36	1.00	Confirmed
NDVI	6.89	6.90	4.82	9.89	1.00	Confirmed
GeoMorphology	5.98	5.96	4.09	7.57	0.98	Confirmed
LUMod	5.81	5.75	3.52	8.04	0.98	Confirmed
DistRoad	5.77	5.70	2.48	10.55	0.97	Confirmed
Lineament	5.22	5.17	1.54	8.24	0.92	Confirmed
Slope	3.05	3.09	0.74	5.69	0.63	Confirmed
DistRiver	1.99	2.11	−0.24	4.16	0.15	Rejected
BWS	1.70	1.60	−0.74	4.55	0.11	Rejected
TWI	1.70	1.60	−0.74	4.55	0.11	Rejected
TPI	1.02	0.87	−0.84	2.75	0.02	Rejected
SPI	0.76	0.85	−0.22	2.15	0.01	Rejected
ProCurvature	0.74	1.07	−0.75	2.01	0.00	Rejected
Aspect	0.22	0.22	−1.56	1.74	0.00	Rejected
LULC	0.17	0.20	−2.36	2.32	0.00	Rejected
HBSE	−0.99	−1.38	−2.11	0.99	0.00	Rejected
GMIS	−1.46	−1.62	−3.17	0.52	0.00	Rejected

### 4.6 GRACE analysis

**Fig. 10:**
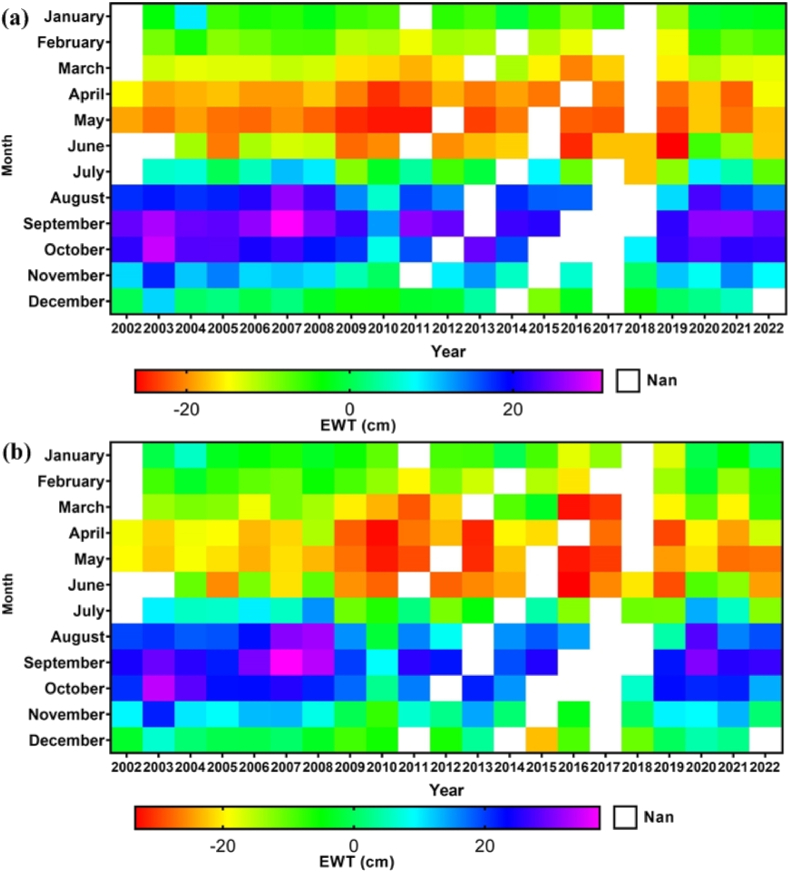
Monthly EWT variation from 2002 to 2022 derived from GRACE (a) CSR and (b) JPL products in Subarnarekha River basin.

As suggested by Scanlon et al. [76]; the utilization of Gravity Recovery and Climate Experiment (GRACE) products for the measurement of equivalent water thickness (EWT) has proven instrumental. This choice is particularly noteworthy due to the substantial enhancement in the spatial localization and amplitude of improved terrestrial Total Water Storage anomalies (TWSA). The generation of GRACE-based EWT heat maps effectively illustrated the regional water balance pattern within the study area. To obtain comprehensive information, data were gathered from the Centre for Space Research (CSR) and the Jet Propulsion Laboratory (JPL), as depicted in Fig. 10 (a) and 10 (b).

The monthly GRACE products were averaged for the area to deliver anomalies in EWT in centimeters during the period from 2002 to 2022. The EWT range of CSR and JPL products was −29.49 to 37.30 cm. The heat maps generated from the GRACE-based EWT data illustrate the regional water balance patterns within the study area (SF.4).

In the early years, water availability in the basin region showed seasonal fluctuations, with levels under the mean from March to June and above the mean from July to November. Analyzing Equivalent EWT dynamics from CSR data, peak EWT occurred in August–October of 2003, 2007, 2011, 2020, and 2021 (30 cm), while the minimum was noted in April–June of 2009, 2010, 2016, and 2018 (−15 cm). Similarly, JPL data revealed a maximum EWT of 30 cm in August–October of 2003, 2007, 2008, and 2020, with a minimum recorded at −15 cm in March–June during 2010, 2013, 2016, and 2017, respectively. These patterns provide insights into temporal variations in water availability.

Analysis of heat maps for the study area indicates that in the earlier years (2002–2007), water availability was higher from August to October. However, in recent years (2010–2019), there's been a decreasing trend in EWT during April to June. This decline may stem from reduced precipitation and the influence of climate change, marked by elevated daily and monthly temperatures. A parallel study by Salehie et al. [77] using GRACE solutions also highlighted a decline in water resources, ranging from 0.04 to 0.08 cm per year, within the Aral Sea's delta basin.

### 4.7 Scope and limitation of the models

The ranking of individual factors can sometimes be criticized due to the need for a fixed pattern. However, grouping individual parameters with similar properties and proximity into primary criteria can reduce the overlap of influence from individual factors on GWPZ. The range of individual factors under a particular class may have localized effects. For instance, this study area's precipitation ranged from 1200 mm to 1800 mm, which will not hold up well in other study areas. The cumulative effect of individual factors under primary criteria can determine the degree of suitability for the final mapping [78]. Incorporating machine learning models (e.g., RF, XGBoost, SVM), advanced cloud pltaform (e.g., Amazon web sservices (AWS), Microsoft azure, Climate engine (CE), OpenEO) and metaheuristic optimization techniques (e.g., genetic algorithm, and particle swarm optimization) can introduce new modeling approaches for GWPZ mapping [18,[48], [79], [80], [81], [82]]. Cloud platforms support the rapid assessment of numerous individual factors and enable the incorporation of large volumes of data. Modeling with climate change scenarios may provide a better understanding of future GWPZ in the study area. Indian meteorological data on precipitation with grid-based distribution may offer improved precipitation zoning compared to CHIRPS.

## 5 Conclusion

This study commenced a novel and highly effective approach for assessing groundwater resources in the Subarnarekha River basin, India, using a hybrid MCDM model. The integration of FuzzyAHP, FuzzyDEMATEL, and LR MCDM models, along with GIS and RS techniques through the GEE cloud platform. The average of all models revealed that approximately 14.74 % of the study area had a very high potential for groundwater occurrences, primarily in the southern and northern parts of the basin. Conversely, the middle part of the basin exhibited very low potential, covering approximately 10.71 % of the area. The hybrid MCDM model was rigorously validated using real-field yield data, achieving an accuracy level of 77 % and an impressive AUC score of 78 %. This hybrid MCDM proved to be a unique and robust method for identifying and quantifying GWPZ in the Subarnarekha River basin. The RF and Boruta algorithms also highlighted the significance of factors, such as soil texture, elevation, slope, precipitation, soil moisture, and runoff in GWPZ modeling, in conjunction with other influencing variables. An alarming trend was observed during the study regarding the availability of water resources, which had decreased during 2010–2019 compared to the 2002–2007 period in our study area.

The study's methodology was detailed and practical, which can be utilized for analyzing groundwater inquiry in other areas. It has enormous potential for boosting agricultural productivity and enhancing irrigation systems. These findings underscore the advantages of leveraging earth observation products for diverse applications, including groundwater stress monitoring, informed land-use planning, accurate water budget estimation, comprehensive water health monitoring, effective disaster management, sustainable groundwater preservation, and a reliable freshwater supply. Further, the study employed extensive groundwater yield data and aquifer information through the advanced hybrid machine learning and deep learning model to improve accuracy.

## Data availability statement

The associated data is currently not deposited in any publicly available repository. Nevertheless, the data will be promptly provided upon request.

## CRediT authorship contribution statement

**Chiranjit Singha:** Writing - original draft, Methodology, Formal analysis, Data curation. **Kishore Chandra Swain:** Writing - original draft, Validation, Supervision, Methodology, Investigation, Formal analysis, Conceptualization. **Biswajeet Pradhan:** Writing - review & editing, Validation, Supervision, Formal analysis, Conceptualization. **Dinesh Kumar Rusia:** Writing - review & editing, Validation, Supervision, Data curation, Conceptualization. **Armin Moghimi:** Writing - review & editing, Writing - original draft, Visualization, Validation, Supervision, Software, Methodology, Conceptualization. **Babak Ranjgar:** Writing - review & editing, Writing - original draft, Validation, Methodology, Investigation, Data curation.

## Declaration of competing interest

The authors declare that they have no known competing financial interests or personal relationships that could have appeared to influence the work reported in this paper.
