# Supplementary material for: Mapping groundwater potential zone in the subarnarekha basin, India, using a novel hybrid multi-criteria approach in Google earth Engine
Source: Heliyon. 2024 Jan 7;10(2):e24308. doi: 10.1016/j.heliyon.2024.e24308 (PMC10825493; doi:10.1016/j.heliyon.2024.e24308)
Supplement: Multimedia component 1 [file mmc1.docx]

**Mapping Groundwater Potential Zone in the Subarnarekha Basin, India, using a Novel Hybrid Multi-Criteria Approach in Google Earth Engine**

**Supplementary Text.1**

**Groundwater influencing factors**

All the factors impacting groundwater are depicted in Supplementary Figure 1. Their respective descriptions are elaborated upon in the following subsections.

**Hydrologic factor**

Geo-hydrological factors directly impact any region's discharge-recharge of surface and sub-surface water. These hydrological factors consist of seven parameters based on their relevance: Topographic wetness index (TWI), Stream power index (SPI), Drainage density, Precipitation, Distance from rivers, Total runoff (RO), and Groundwater stress.

TWI describes spatial moisture patterns in a region due to water accumulation due to topographic variability and runoff effects. Low TWI values are associated with steep slopes, indicating lower water availability and higher runoff, whereas low-laying depressed regions have high TWI values, which enhance groundwater storage capacity. TWI values in the study area were found to vary from 6.45 to 24.73. Another crucial groundwater potential factor, SPI, expresses streams' erosive power. SPI and TWI values can be calculated using the following equations (Eq. 1 & 2):

$SPI=A_{s}\times tan\beta$ (1)

$TWI=\left( \frac{A_{s}}{tan\beta} \right)$ (2)

where $A_{s}$ represents the cumulative surface of the ascending slope, and $\beta$ indicates the gradient of the slope.

Rainfall data were acquired from the Climate Hazards Group InfraRed Precipitation with Station Data (CHIRPS) with 0.05° resolution for the last 20 years (1981–2020) from four rain gauges stations located within ad near the river basin region on the Google Earth Engine Platform. The interpolation method used for mapping annual rainfall patterns was Kriging. They conducted in ArcGIS 10. Drainage density is inversely correlated to. Hence, low-density values favored high groundwater potential and were allocated higher weights. Drainage density was computed using the line density tool in the ArcGIS arc toolbox. They were using Eq. 3.

$D_{d}=\frac{\sum_{i=1}^{n} L_{i}}{A}$ (3)

Where $\sum_{i=1}^{n}$describes the total length of the drainage network (L), A denotes the total area of the river basin (L^2^), and n represents the number of drainage networks in the sub-river basin (-).

Rivers and their branches may either gain water from groundwater or lose water regarding recharge to groundwater storage. So, the distance to a river significantly affects groundwater recharge potentiality. Areas within 500m of a river are most promising for effective infiltration due to new alluvial soil. In comparison, areas beyond > 500m from the river may have less appropriate permeable formations. Using the line density tool in ArcGIS, the Euclidean distance method determined the distance from the river. Surface runoff can affect changes in groundwater storage rates in an area. Runoff is inversely correlated with the recharge rate. Low surface runoff and high percolation rates help identify areas with good groundwater potential. Total runoff was estimated using the GLDAS forcing Model for the last 17 years (2003-2020) in the area. The baseline water stress in the study area was calculated using the global hydrological PCR-GLOBWB 2 run model with Eq. 4. Baseline water stress measures the ratio of total water withdrawals to available renewable surface and groundwater supplies. Higher values specify more struggle among users to lower groundwater potential.

$ws_{m,y,b,ols10}=\frac{ww_{m,y,b,ols10}}{max\left( Q_{m,y,b,ols10} \right)-wn_{m,y,b,ols10}}$ (4)

Where,$wS_{m,y,b,ols10}$ represents water stress per month, per year, per sub-basin in (-)$ww_{m,y,b,ols10}$ denotes gross (consumptive plus non-consumptive) total withdrawal per month, per year, per sub-basin in (m/month)

$Q_{m,y,b,ols10}$ represents available water per month, per year, per sub-basin in (m/month) $wn_{m,y,b,ols10}$ denotes net (consumptive) total withdrawal per month, per year, per sub-basin in (m/month).

Ordinary least square (OLS) regression computed the baseline water stress where a baseline year is 2014 (Eq.5).

$bwS_{m,s}=OLS\left( wS_{m,y,b,ols10} \right)_{2014}$ (5)

Where, $bwS_{m,s}$represents a raw value of baseline water stress per month per sub-basin $wS_{m,y,b,ols10}$ represents water stress per month, per year, per sub-basin (-).

In the study area, the baseline water stress percentage ranges from <10% to >80% based on Aqueduct 2.1.

**Permeability factor**

Permeability factors, such as soil type, soil moisture, soil evapotranspiration (ET), and soil loss, were considered in estimating surface and sub-surface groundwater flow for potential groundwater storage. Soil factors are the most critical parameters for delineating the soil infiltration, percolation, and leaching rate into an aquifer. The soil structure and texture analysis revealed that sandy clay loam and loam soil have outstanding potential for groundwater occurrence in the study area. The soil map was derived from the Food and Agriculture Organization (FAO) harmonized grid world soil database (http://www.fao.org/soils-portal). Further, the soil classes were classified using the Soil and Water Assessment Tool (SWAT) Soil Database. Soil moisture data was derived from the L band Soil moisture active passive (SMAP) dataset for 2015-2020 via the Google Earth Engine (GEE) cloud code editor. In the study area, the high soil moisture values ranging from 17.70 mm - 21.97 mm are associated with a high occurrence of groundwater potential. High soil evapotranspiration indicates shallow groundwater levels, while low ET suggests deeper groundwater levels, especially in dry climatic conditions. In the study area, ET values ranging from 0.000045 – 0.000049 kg m^-2^ s^-1^ represent shallow groundwater depths. The time-averaged map of evapotranspiration (2010-2020) was acquired from the Global Land Data Assimilation System (GLDAS) model and is available in the Engine data catalog. Soil loss is critical in groundwater storage change due to soil regolith formation and depth. High soil erosion rates were observed in areas with steep slopes, bare ground, and unscientific land management practices, accelerating runoff and reducing soil permeability, leading to groundwater table depletion. Soil loss (tons per hectare per year) was estimated using the RUSLE-based Global Soil Erosion Modeling platform (GloSEM) (Borrelli et al., 2013). The Global Soil Erosion map (GeoTIFF raster) at a 25 km resolution was obtained from the European Soil Data Centre (ESDAC).

**Morphometric factor**

Morphometric factors encompass various parameters, including elevation, slope, aspect, profile curvature, ruggedness index, lineament density, topographic position index, lithology, and geomorphology. A digital elevation model (DEM) was extracted from the Shuttle Radar Topography Mission (SRTM) data and then used to derive morphometric factors. Elevation plays a significant role in influencing groundwater prospects. Higher elevations typically indicate a lower potential for groundwater, while lower elevations suggest a higher potential . Groundwater recharge, on the other hand, is affected by slope factors. Gentle slopes facilitate higher percolation rates, whereas steeper slopes result in increased surface runoff. Aspect, another important criterion, indirectly impacts groundwater potential by affecting factors such as flow direction, soil moisture, and the angle of solar radiation incidence, all of which can influence infiltration rates. Profile curvature factors were extracted from the DEM using spatial analysis tools in ArcGIS software. These factors primarily affect flow velocity by causing deceleration and acceleration, divergence, and convergence at the soil surface. The ruggedness index quantifies topographic heterogeneity, ranging from central pixels to edge cells within an area. A higher ruggedness index value corresponds to a more significant elevation and slope, resulting in a lower groundwater potential. In our study, the ruggedness index ranged from 80 to 422. Lineament density is directly proportional to groundwater potential and can be expressed in various ways, such as lineament length density, lineament count density, and lineament cross-point density. We selected lineament density as a factor for estimating groundwater recharge potential. The lineament density data for the Subarnarekha basin region were obtained from the Geological Survey of India, 2019 at a resolution scale of 1:250,000. Lineament density (LD) for groundwater targeting in our study area is characterized by the total length of lineaments within a unit area, as expressed in Eq. 6.

$L_{d}= \frac{\sum_{i=1}^{i} L_{i}}{A}$ (6)

where, $\sum_{i=1}^{i} L_{i}$ indicates the total length of lineaments (L) and A expressed as a unit area (L^2^).

Areas with a lineament density between 0.33 and 0.41 km/km² were categorized as zones with a very high groundwater potential. The topographic position index (TPI) associates the elevation of each pixel in the DEM with the mean height of the surrounding pixels (Eq. 7). As explained, negative and positive values indicate whether a pixel is lower or higher in elevation than the surrounding pixels.

TPI = $\frac{E_{pixel}}{E_{surrounding}}$ (7)

where $E_{pixel}$represents the elevation of the cell, and $E_{surrounding}$is the average elevation of the neighboring pixels.

The lithological materials influence Soil porosity and permeability, affecting groundwater movement, aquifer stability, and potential efficiency. Geomorphological features, representing different landforms, also play a role in groundwater occurrences. Various geomorphic types, such as floodplains and alluvial areas, have higher aquifer storage capacity than highly dissected structural plains and denudational hills. The lithological features in the study area exhibit variability, and areas with high to very high groundwater occurrences are associated with materials such as sand, silt, clay, yellowish-brown fine sand, clay, gravel beds, volcanic agglomerates, calcareous concrete, lateritic soil, laterite, limestone, impure marble, calc-silicate rocks, limestone, grit, arkose, conglomerate, calc schist, and dolomite. Lithological data and the geomorphology information in the Geotiff file (1:250,000 WGS84) were acquired from the Geological Survey of India, 2019.

**Terrain distribution**

Terrain distribution parameters significantly influence the movement of surface and sub-surface runoff, groundwater recharge, and evapotranspiration. Terrain distribution serves as a valuable indicator for identifying potential aquifer recharge zones, which can lead to higher infiltration rates. This study utilizes several factors, including Land Use/Land Cover, Normalized Differential Vegetation Index (NDVI), and The Global Human Modification of Terrestrial System (GHMTS). NDVI is crucial in assessing vegetation types and density, which depend on moisture conditions and promote soil infiltration—a key indicator of groundwater potential. Consequently, higher NDVI values are given greater significance. In the study area, NDVI values range from -0.02 (indicating water cover) to 0.63 (corresponding to forested areas). Median NDVI values were calculated per pixel by normalizing the difference between the red and NIR bands using Sentinel 2 level-1C images with a 10 m spatial resolution. These images were acquired from January 1, 2021, to December 31, 2021, via the European Space Agency (ESA) on the GEE cloud server (https://code.earthengine.google.com) (Eq. 8).

$NDVI=\frac{NIR-RED}{NIR+RED}$ (8)

The Land Use/Land Cover map for the year 2020 was generated using Sentinel-2 and Sentinel-1 data in the Google Earth Engine code editor. This high-resolution map, with a spatial resolution of 10 meters, was obtained from the WorldCover Land Use/Land Cover (LULC) products provided by the European Space Agency (ESA) project (<https://esa-worldcover.org/en>). The distribution of groundwater is significantly influenced by the pattern of land use. Areas with more excellent vegetation coverage promote the expansion of aquifer layers and enhance infiltration compared to sparsely vegetated and barren lands. Various human activities, such as urban development, transportation, agriculture, electrical infrastructure, energy production, mining, and population growth, have led to alterations in topography and changes in structural slopes, potentially impacting water resources and terrestrial ecosystems. The Global Human Modification of Terrestrial Systems v1 data provides information about different human modification stressors, where a value of '0' indicates no modification and '1' signifies high modification. GHMTS data were obtained in raster format from https://sedac.ciesin.columbia.edu/data/set/lulc-human-modification-terrestrial-systems.

**Anthropogenic interference**

Anthropogenic interference parameters include population density, Global Human Built-Up and Settlement Extent (HBASE), Global Man-Made Impervious Surface (GMIS), and distance from roads, which significantly impact groundwater potential recharge. Parameters such as distance to roads, built-up area, population density, and impervious surfaces play crucial roles in groundwater prediction. This study classified distances to roads into five classes using the Euclidean distance tool in ArcAGIS, with intervals of 500 meters. The road network vector data were obtained from OpenStreetMap at <https://www.openstreetmap.org/export#map=6/22.431/83.035>. Population growth and the rapid expansion of built-up areas can increase water demand for groundwater extraction. They may result in creating more impervious surface areas, affecting the infiltration rate into groundwater storage. The GMIS raster layer represents the percentage of imperviousness, ranging from 0 to 100%. In this study, higher imperviousness indicates lower infiltration capacity, putting more stress on aquifer storage. The HBASE layer was created using the random forest algorithm, with 201 indicating HBASE and 200 representing non-HBASE areas. Population density, GMIS, and HBASE datasets were estimated using Landsat v1 data from the NASA Socioeconomic Data and Applications Center (SEDAC), based on Geotiff raster layers, using the Google Earth Engine (GEE) platform.

| 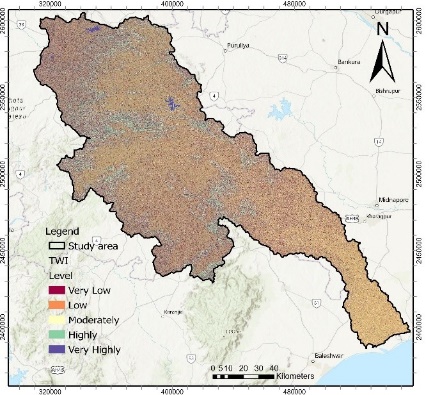 | 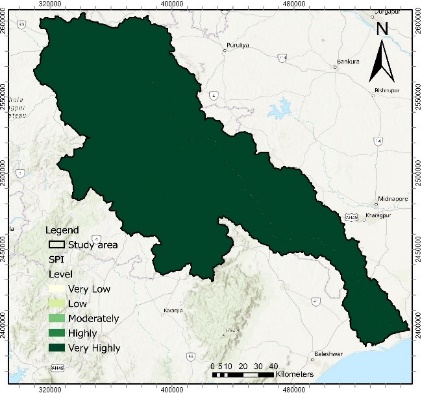 | 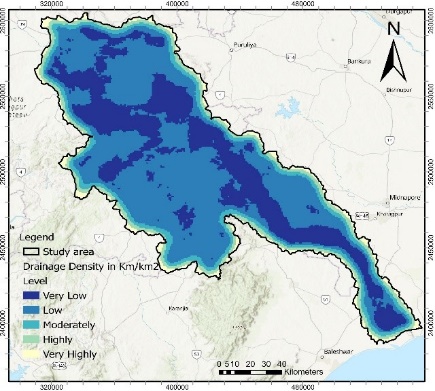 |
| --- | --- | --- |
| (a) | (b) | (c) |
| 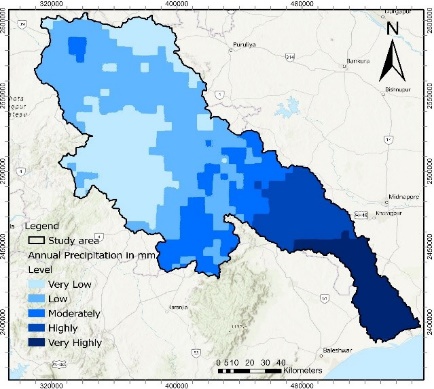 | 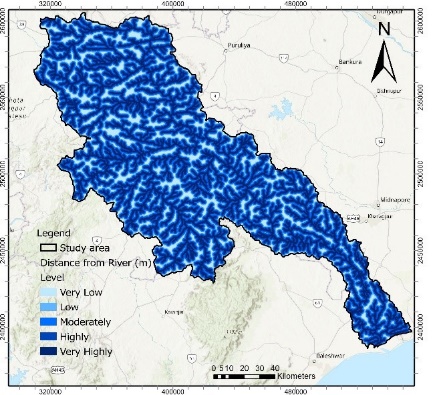 | 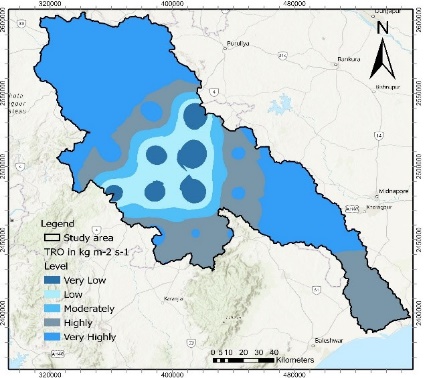 |
| (d) | (e) | (f) |
| 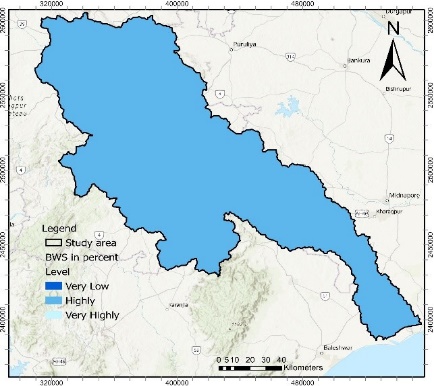 | 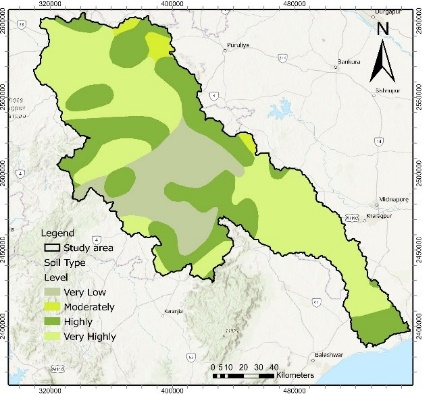 | 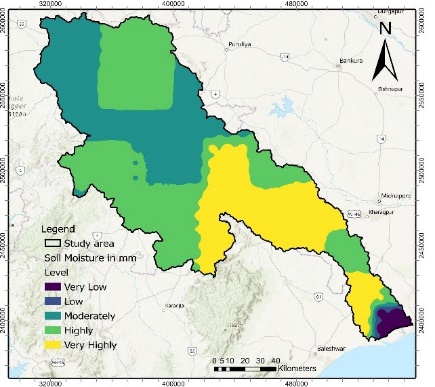 |
| (g) | (h) | (i) |
| 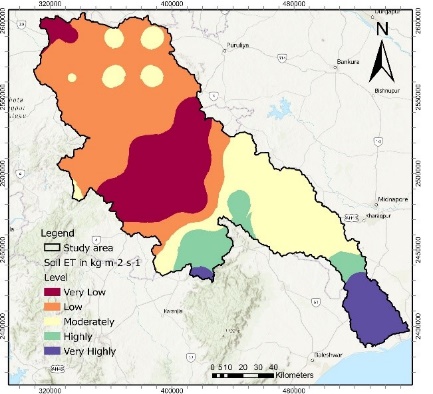 | 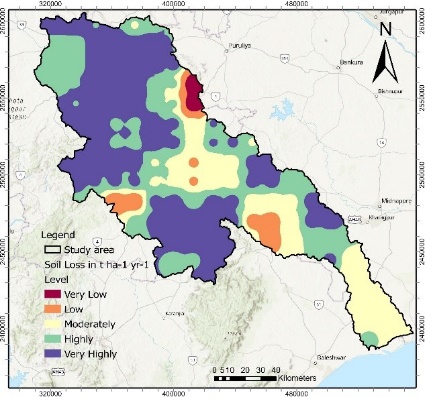 | 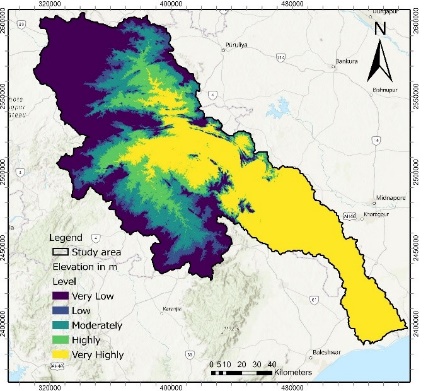 |
| (j) | (k) | (l) |
| 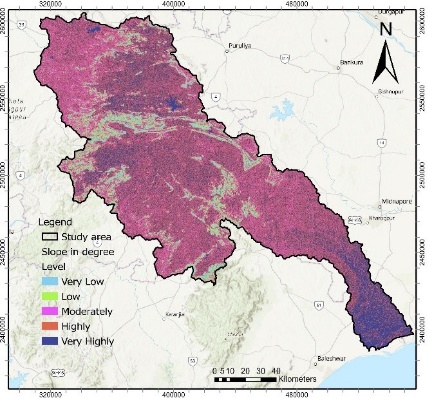 | 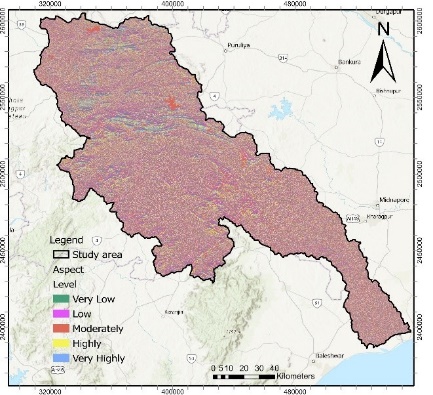 | 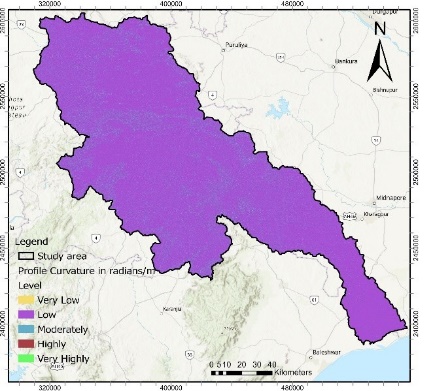 |
| (m) | (n) | (o) |
| 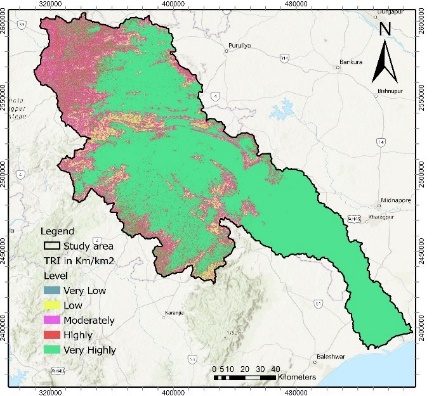 | 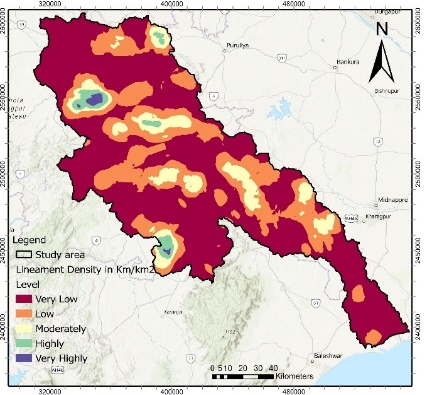 | 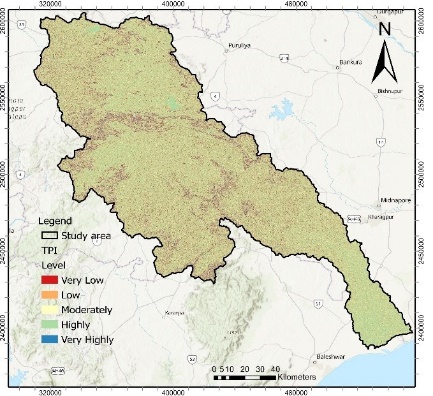 |
| (p) | (q) | (r) |
| 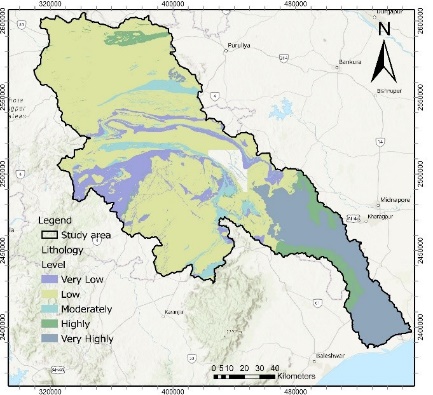 | 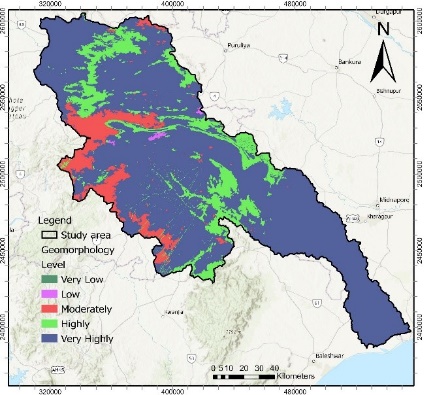 | 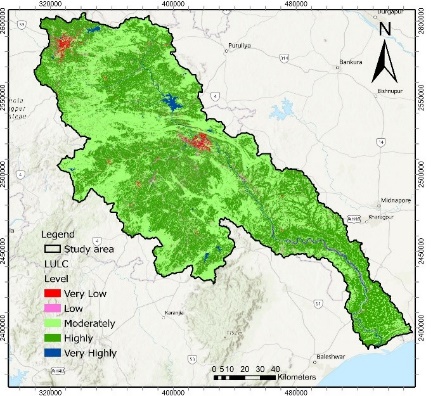 |
| (s) | (t) | (u) |
| 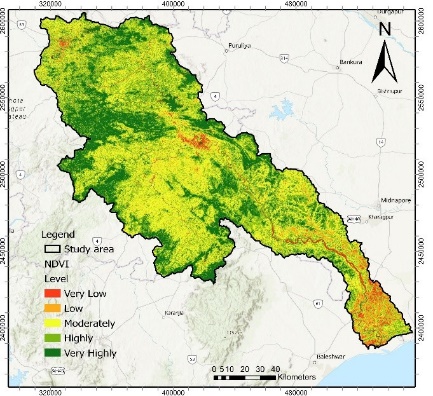 | 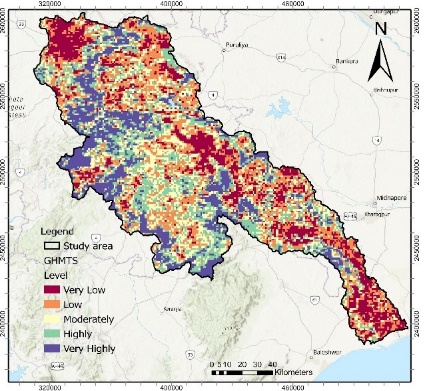 | 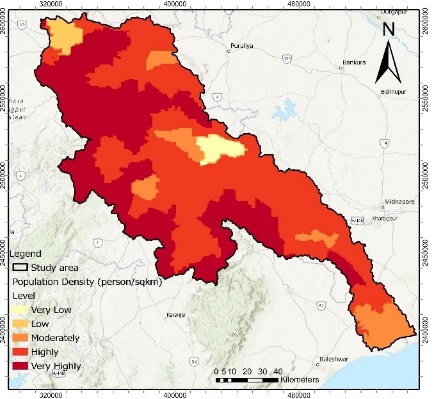 |
| (v) | (w) | (x) |
| 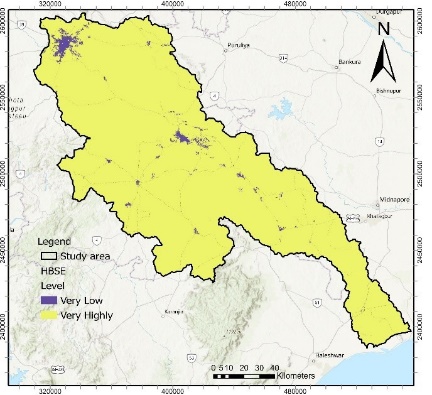 | 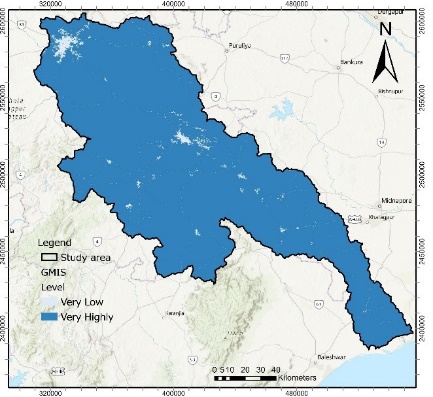 | 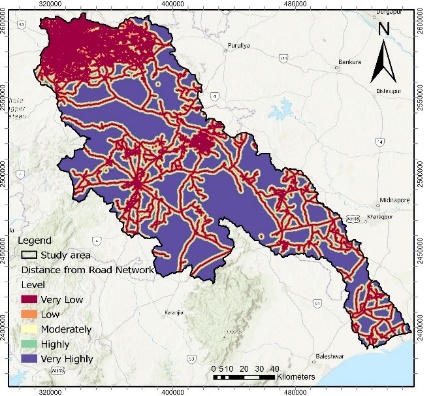 |
| (y) | (Z) | (aa) |

**Supplementary Figure 1.** Distribution map of sub factors; I) Hydrologic subfactor maps (a)topographic wetness index (TWI), (b) stream power index (SPI), (c) drainage density, (d) precipitation, (e) distance from rivers, (f) total runoff (RO) (g) groundwater stress; II) Permeability subfactor maps (h) soil type, (i) soil moisture, (j) soil evapotranspiration (ET), (k) Soil loss; III) Morphometric sub factor maps (l) elevation, (m) slope, (n) aspect, (o) profile curvature, (p) ruggedness index, (q) lineament density, (r) topographic position index (TPI), (s) lithology, (t) geomorphology; IV) Terrain distribution subfactor maps (u) land use/land cover, (v) normalized differential vegetation index (NDVI), (w) global human modification of terrestrial system (GHMTS); V) Anthropogenic interference sub factor maps (x) population density, (y) global human built-up and settlement extent (HBASE), (z) global artificial impervious surface (GMIS), (aa) distance from roads.


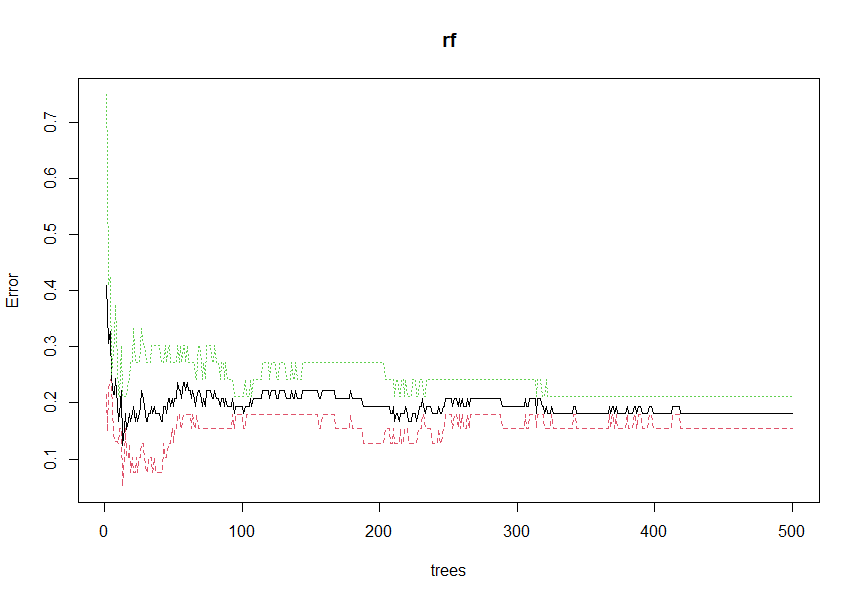


**Supplementary Figure 2:** Relationship between the number of trees and OOB error

*l*

$$M_{\tilde{M}}$$

*M*

*1.0*

*0.0*

*u*

*m*

$$M^{r(y)}$$

$$M^{l(y)}$$

**Supplementary Figure 3:** Triangular fuzzy number (TFN) set for GWPZ mapping

**Supplementary Table 1:** Transforming linguistic variables into corresponding TFNs

| Abbreviations | Linguistic Preferences | Corresponding TFNs |
| --- | --- | --- |
| VHL | Very high influence | (0.75, 1, 1) |
| HI | High influence | (0.5, 0.75, 1) |
| L | Low influence | (0.25, 0.5, 0.75) |
| VL | Very low influence | (0, 0.25, 0.5) |
| NO | No influence | (0, 0, 0.25) |
